# An In Vivo and In Silico Approach Reveals Possible Sodium Channel Nav1.2 Inhibitors from *Ficus religiosa* as a Novel Treatment for Epilepsy

**DOI:** 10.3390/brainsci14060545

**Published:** 2024-05-27

**Authors:** Aqsa Ashraf, Abrar Ahmed, André H. Juffer, Wayne G. Carter

**Affiliations:** 1 Faculty of Pharmacy, Punjab University College of Pharmacy, University of the Punjab, Lahore 54590, Pakistan; aksa.ashraf95@gmail.com; 2 Biocentre Oulu (BCO) and Faculty of Biochemistry and Molecular Medicine (FBMM), University of Oulu, 90570 Oulu, Finland; andre.juffer@oulu.fi; 3 Clinical Toxicology Research Group, School of Medicine, University of Nottingham, Royal Derby Hospital Centre, Derby DE22 3DT, UK

**Keywords:** epilepsy, *Ficus religiosa*, 6-C-glucosyl-8-C-arabinosyl apigenin, maximal electroshock induced seizure model, molecular docking, Na^+^ channel Nav1.2, pelargonidin-3-rhamnoside

## Abstract

Epilepsy is a neurological disease that affects approximately 50 million people worldwide. Despite an existing abundance of antiepileptic drugs, lifelong disease treatment is often required but could be improved with alternative drugs that have fewer side effects. Given that epileptic seizures stem from abnormal neuronal discharges predominately modulated by the human sodium channel Nav1.2, the quest for novel and potent Nav1.2 blockers holds promise for epilepsy management. Herein, an in vivo approach was used to detect new antiepileptic compounds using the maximum electroshock test on mice. Pre-treatment of mice with extracts from the *Ficus religiosa* plant ameliorated the tonic hind limb extensor phase of induced convulsions. Subsequently, an in silico approach identified potential Nav1.2 blocking compounds from *F. religiosa* using a combination of computational techniques, including molecular docking, prime molecular mechanics/generalized Born surface area (MM/GBSA) analysis, and molecular dynamics (MD) simulation studies. The molecular docking and MM/GBSA analysis indicated that out of 82 compounds known to be present in *F. religiosa*, seven exhibited relatively strong binding affinities to Nav1.2 that ranged from −6.555 to −13.476 kcal/mol; similar or with higher affinity than phenytoin (−6.660 kcal/mol), a known Na^+^-channel blocking antiepileptic drug. Furthermore, MD simulations revealed that two compounds: 6-C-glucosyl-8-C-arabinosyl apigenin and pelargonidin-3-rhamnoside could form stable complexes with Nav1.2 at 300 K, indicating their potential as lead antiepileptic agents. In summary, the combination of in vivo and in silico approaches supports the potential of *F. religiosa* phytochemicals as natural antiepileptic therapeutic agents.

## 1. Introduction

Epilepsy is a neurological disease characterized by unpredictable and recurrent convulsive events, termed epileptic seizures [1]. Such unhinged interruptions of normal brain function are precipitated due to a hypersynchronous discharge of neuronal cells. This orchestration of the neuronal synchronization pathway is governed by the ion channels embedded in the neuronal membranes including Na^+^, K^+^, and Ca^2+^ channels [2]. Voltage-gated sodium channels (VGSCs) are chiefly reported to mediate intrinsic neuronal excitability via the conduction of Na^+^ ions across cell membranes during cell membrane depolarization. Hence, anomalous sodium channel activity is responsible for several channelopathies varying from chronic pain to epilepsy [3,4].

Mammalian VGSCs are composed of a pore-forming α subunit and one or two smaller β (auxiliary) subunits and there are nine α-subunit isoforms; Nav1.1 to Nav1.9, with multifarious expression patterns and physiological roles [5]. The Nav α-subunits are approximately 2000 amino acids in length, assembled into four domains (D1–D4), and each domain is comprised of six transmembranous α-helices [6]. Collectively, two of the α-helices from each of these domains contribute to the formation of the pore whereas the other four α-helices from each domain form the voltage sensor [6]. The altered expression and existence of mutations in the genes that code for the Nav α-subunits, including SCN1A, SCN1B, SCN2A, SCN3A, and SCN8A contribute to epileptic diseases [7]. Compounds that inhibit sodium channels attenuate the Na^+^ current and therefore prevent the repetitive firing of neurons and stabilize the neuronal membranes, limiting aberrant seizure activity [3]. Consequently, many pharmacotherapies for epilepsy, including carbamazepine, phenytoin, and lamotrigine, are inhibitors of VGSC function [7].

Conventional pharmacological therapy for epilepsy with drugs including phenytoin, carbamazepine, and phenobarbital has been associated with major adverse drug effects including idiosyncratic reactions, cognitive impairment, and teratogenicity [8,9,10,11,12,13]. Additionally, over one-third of clinic-based epileptic patients are refractory to pharmacotherapy [14]. One mechanism that has been postulated to account for refractory epilepsy is from polymorphic variants in the VGSC genes, including SCN2A, the gene for human Nav1.2 [15,16,17,18]. Hence, there is an unmet need for the development of safer and more specific anticonvulsant drugs and novel drugs that may be able to treat drug-resistant epilepsy. 

Medicinal plants have been historically projected as the fountainheads of new lead compounds for pharmaceutical development [19,20]. Phytochemicals with bioactivity can provide complementary compounds to those produced by chemical synthesis [19]. *Ficus religiosa* (family Moraceae) is one such medicinal plant recognized for its phytochemical diversity, therapeutic, and mythological and religious significance. It has widespread distribution, thriving among tropical Asian countries including India, Pakistan, Nepal, and Bangladesh [21]. In addition to a plethora of its curative properties for diseases such as bleeding disorders, digestive impairments, rheumatism, topical disorders, urinary obstructions and antidiabetic, antioxidant and anti-amnesic profiles, the plant has been postulated to possess significant antiepileptic activity in ethnomedicinal scriptures and contemporary research studies [22,23,24,25,26,27]. These therapeutic properties have been attributed to the presence of secondary metabolites including campesterol, serotonin, lanosterol, methyl oleanolate, stigmasterol, β-sitosterol, lupin 3-one, bergenin, amides, and caffeic acid, as well as a number of flavonoids [26,28,29,30]. 

Drug discovery from medicinal plants utilizes various phytochemical, molecular, and biological assays often used in conjunction with advanced computer-aided modeling techniques [31]. Molecular docking, molecular mechanics/generalized Born surface area (MM/GBSA), and molecular dynamics (MD) simulation are examples of such computational (in silico) techniques employed for lead compound discovery and design [32,33,34]. 

Animal models for epilepsy aim to mimic the pathophysiology and phenomenology of human epilepsy but this is not completely feasible given the heterogeneous and complex nature of seizures in humans [35]. The maximal electroshock seizure (MES) test has been employed as a model for generalized tonic and/or clonic seizures [35,36]. The MES test has provided predictive validity for antiepileptic drugs but other models such as the pentylenetetrazol (PTZ) test or the 6 Hz seizure test have proved useful for generalized absence or partial seizures [35,36,37,38,39]. Indeed, marketed antiepileptic drugs such as phenytoin and carbamazepine, which are known to inhibit VGSCs, were tested using these models [35,38,40,41,42].

In this study, we first assessed the antiepileptic potential of *Ficus religiosa* phytocompounds against induced epileptic seizures employing the MES test, followed by the application of high-efficiency computational techniques, including molecular docking, MM/GBSA, and molecular (MD) studies to explore their potential as blockers of the human Na^+^ channel Nav1.2. 

## 2. Materials and Methods

### 2.1. Collection of Plant Material, Extract Preparation, and Fractionation

Whole plant parts of *F. religiosa* were collected from the University of the Punjab, Lahore, Pakistan (31°29′ north latitude, 74°17′ east longitude). The procured plant specimen was authenticated by Prof. Dr. Zaheer-ud-din Khan from the Department of Botany, GC University Lahore (GCUL), and deposited at the university’s herbarium with voucher number 3862. The collected plant material was shade-dried for seven days at ambient temperature with adequate ventilation and protected from direct sunlight. Leaves, twigs, branches, and adventitious roots were all used for preparation of plant extracts in order to be representative of the whole plant. The dried plant parts were ground to a coarse powder and then extracted by maceration with ethanol (95%) for ten days. The ethanolic crude extract was then filtered using Whatman filter paper No. 1, followed by concentration using a vacuum rotary evaporator. This dried plant extract was then dispersed in distilled water and fractionated with petroleum ether (PE, non-polar solvent) and ethyl acetate (EA, semi-polar solvent) to segregate the plant constituents based on their inherent polarity profiles. Each fraction (PE-Fr and EA-Fr) was then subjected to drying under reduced pressure to remove the admixed solvents and the dried extracts were stored for subsequent use.

### 2.2. Experimental Animal Studies

Twenty-four twelve-week-old male Swiss albino mice weighing 30 ± 5 g were purchased from the University of Veterinary and Animal Sciences (UVAS), Lahore, Pakistan. The procured mice were randomly sorted into 4 different groups (6 mice per group), accommodated in polycarbonate cages, and housed under controlled conditions, i.e., at room temperature (23 ± 2 °C), with a relative humidity of 55 ± 5%, a 12/h light/dark cycle, and ad libitum access to food and water. Mice were acclimatized in the laboratory for 2 h before testing, with all experimental tests performed on the same day to avoid any periodic variation in convulsive susceptibility. All experimental investigations conformed to the institution’s animal care and use committee (IACUC) guidelines, as approved by The Punjab University Institutional Ethics Review Board, under receipt number 1476 on the 29th of September 2022.

#### 2.2.1. In Vivo Study Design

Experimental Group 1 was a negative control group administered with 0.9% saline (manufactured by Otsuka Pakistan, Ltd., Balochistan, Pakistan), which was also used as the vehicle to dilute the test extracts. Group 2 was a positive control group that received the standard drug, phenytoin (manufactured by ATCO laboratories, Pakistan). Group 3 was administered the petroleum ether *F. religiosa* (PE-Fr) extract and Group 4 the ethyl acetate *F. religiosa* extract (EA-Fr). 

Similar to other published methods, treatments were undertaken 30 min before seizure testing [43,44]. Group 1 was administered 0.9% NaCl solution intraperitoneally (i.p.), and Group 2 received 25 mg/kg phenytoin in a volume of 10 mL/kg (i.p.). Since 400 mg/kg has been documented as a therapeutic dose of *F. religiosa* [29], the plant extracts PE-Fr (400 mg/kg) and EA-Fr (400 mg/kg) in a volume of 10 mL/kg were administered orally to Group 3 and Group 4, respectively, 1 h before testing.

#### 2.2.2. Maximal Electroshock (MES)-Induced Seizure Test

The MES convulsion model was used to assess the anticonvulsant potential of the plant extracts. Electroshock (50 mA, for a duration of 0.2 s) was used to induce convulsions in each mouse using a rodent shocker, via saline-wet trans-auricular electrodes. Abolition of the tonic hind limb extension (THLE) was set as an indicator of the inhibition of MES-induced seizure spread [45]. The results were video recorded and then statistically compared with control and standard groups for the determination of anticonvulsant activity.

#### 2.2.3. Statistical Analysis

Results were tabulated as means ± SD using GraphPad Prism 8.0.1 (https://www.graphpad.com/). Comparisons between different experimental groups were made using one-way analysis of variance (ANOVA) followed by post hoc Dunn’s test. Differences were considered significant when *p* < 0.05.

### 2.3. In Silico Studies

#### 2.3.1. Preparation of Protein

**Figure 1:**
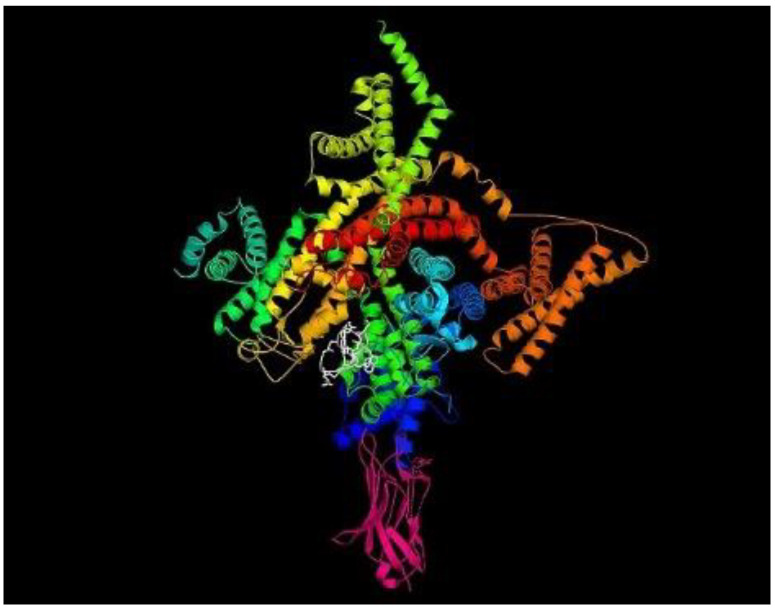
Cryo-EM structure of human sodium channel Nav1.2 (multi-colored) bound to the inhibitory ligand μ-conotoxin KIIIA (tagged in white) in the presence of the auxiliary subunit β2 as shown in pink (Nav1.2-β2-KIIIA with PDB ID: 6J8E).

A cryo-electron microscopy structure of Nav1.2-β2 complex (accession ID: 6J8E) was obtained from the RCSB-Protein Data Bank (PDB) (https://www.rcsb.org/). The three-dimensional structure of the target protein is shown in Figure 1.

Since the raw PDB file consisted of the co-crystallized ligand μ-conotoxin KIIIA, conjoined sodium ion, and bulky carbohydrate chains, with no information on formal atomic charges and bond orders, protein preparation was undertaken to impart chemical correctness to the parent protein structure. The obtained PDB structure was therefore refined using Protein Preparation Wizard presented in the Maestro v13.2 Schrödinger, LLC, 2022.1 software package. This is required in order to produce a well-optimized protein structure, which is a prerequisite for accurate docking calculations to be made using the molecular docking program, Glide (Grid-based Ligand Docking with Energetics by Schrödinger, LLC, 2022.1 software package). Glide utilizes a set of hierarchal filters to identify the spatial fit of each ligand compound within the active site of the target protein structure. Hence, the Protein Preparation Wizard was used to remove the co-crystallized ligand μ-conotoxin KIIIA and the attached sodium ion, missing residues were added, selected chains were provided with polar hydrogens to satisfy their valences, formal charges were adjusted, and proper bond orders were assigned. The structure was protonated to a pH of 7.0 and minimized using the Optimized Potentials for Liquid Simulations (OPLS-2005) force field. The protein structure was thus converted into an optimized configuration that conforms to the requirements for molecular docking studies.

#### 2.3.2. Ligand Selection and Preparation

Through an extensive literature search, 82 phytochemicals reported from *Ficus religiosa* were investigated as prospective Nav1.2 sodium channel blockers to validate their antiepileptic binding potential [46,47,48,49]. Structures of the prospective ligands were drawn using the Maestro v13.2 Schrödinger, LLC, 2022.1 software package. For accurate docking results, these ligands were also structurally optimized before being applied to the Glide software. This was performed using LigPrep, Schrödinger’s in-built feature for ligand preparation. LigPrep performed a series of tasks including the correction of bond orders and bond lengths, optimization of ring conformations, and correction of chirality for all input structures. Undesired structures were eliminated and possible ionization states for each ligand were generated using the built-in Epik module, at a target pH of 7.0. Thereafter, the respective ligands were energy-minimized using the OPLS-2005 force field. These low-energy three-dimensional optimized ligand structures were then used for molecular docking.

#### 2.3.3. Molecular Docking

Following the structural refinement of each ligand and the target protein (Nav1.2), the active site of Nav1.2 was predicted. This was performed using the Glide Receptor Grid Generation panel by removing the co-crystallized ligand, μ-conotoxin KIIIA, that was affixed at the binding pocket of Nav1.2, thereby revealing x, y, and z coordinates. Using the defined receptor grid, docking calculations were then run using Glide Extra Precision (XP-visualizer module). By implementing the protocols of flexible docking, XPGlide created multiple spatial orientations of each ligand relative to the active site of Nav1.2 and performed a heuristic high-throughput virtual screening (HTVS), weeding out the ligand poses that displayed unfavorable energies and predicting the most favorable conformational pose of every ligand. The selected ligand poses were then minimized using the OPLS-AA forcefield followed by further ranking of the best-bound energy minimized ligand poses based on their binding affinities with Nav1.2, as computed through the docking score [50]. Thereafter, the ligand conformations with higher and comparative negative docking scores with the antiepileptic drug, phenytoin, used as the reference ligand, were tabulated in rank order and further analyzed using molecular mechanics/generalized Born surface area (MM/GBSA) studies.

#### 2.3.4. Molecular Mechanics/Generalized Born Surface Area (MM/GBSA) Simulation

MM/GBSA analysis was conducted using the Prime application of Maestro v13.2 Schrödinger, LLC, 2022.1 software package, to calculate the binding free energies (∆*G_bind_*) of the top hits from the docking analysis. These docked complexes were firstly minimized using the OPLS-2005 force field, which also evaluates the binding free energies of the optimized endpoints of the system; free target protein, the free ligand, and the protein–ligand interacting complex, using the following formula:

$$∆G_{bind}=G_{complex}-G_{protein}-G_{ligand}$$

where the free energy (*G*) of each system is computed using the formula:

$$G=E_{MM}+G_{solv}-TS$$

in which, EMM refers to the total molecular mechanics energy corresponding to the component electrostatic and van der Waals energies, *G_solv_* corresponds to the solvation-free energy with regard to polar and non-polar contributions, calculated using the implicit solvation model; generalized Born continuum solvent model, and solvent-accessible surface area (SASA) model, respectively. Whereas TS refers to the product of absolute temperature (*T*) and entropy (*S*), calculated from the normal mode analysis of harmonic frequencies of each system.

#### 2.3.5. Molecular Dynamics (MD) Simulation

MD simulation was used to provide a model system that accounts for the thermodynamical, structural, and kinetic profiles of a drug target biological system [51,52]. An insight into the dynamic configurational character of such a system is obtained through a time-dependent evaluation performed in a computationally built simulation box, where the interatomic contributions are described by the Newtonian laws of motion. The simulations thus proceed to identify ligand–protein complexes that are energetically stable to be used in rational drug design [51]. To estimate the interactive stability of the best-docked protein–ligand complexes, MD simulation studies were employed using Desmond–Maestro v13.2 Schrödinger, LLC, 2022. Since the software provides high-performance algorithms as its inbuilt feature, default settings were applied [53]. To mimic the aqueous environment, an equilibrated orthorhombic water box, centered on the mass center of each ligand, was used to solvate the docked complex. Since it is desirable to electrically neutralize a system before running the simulations [54], Na^+^ and Cl− ions were added. Thereafter, the built system was minimized using a Limited-memory Broyden–Fletcher–Goldfarb–Shanno (LBFGS) algorithm and equilibrated to adjust to the applied force field, OPLS3e. While the applied force field accounted for the component interactive forces of the system, simulations were run for 130 ns under an NPT (Normal Temperature and Pressure) ensemble with 300 K temperature, and 1.01325 bar pressure. Afterward, through trajectory mapping of the velocities, energies, and coordinates of the system’s particles, statistical analysis was performed to determine how perfectly a ligand binds to the target’s active site [51]. Root mean square deviation (RMSD) analysis, the backbone amino acids involved in the binding of the docking complex, and thus the stability of the complex, were then determined and have been described below.

#### 2.3.6. Absorption, Distribution, Metabolism, Elimination (ADME) Analysis

SwissADME, a predictive web-based tool, was used as an alternative to resource-consuming experimental procedures to analyze the physiochemical properties, pharmacokinetic parameters, and drug-likeness of the screened compounds. The presence or absence of certain molecular descriptors of the input chemical structures was used as a criterion for the calculation of the potential drug suitability of a compound, as evaluated through an amalgamation of several predictive computational tools. The estimated characteristics of each compound included the following: molecular weight (MW), molar refractivity, lipophilicity (log Po/w), number of hydrogen bond donors and hydrogen bond acceptors, evaluation of bioavailability score, gastrointestinal (GI) absorption, and blood–brain barrier (BBB) permeation.

The one-click interoperability of SwissADME allowed the calculation of the MW and molar refractivity (MR) of the tested compounds through OpenBabel, version 2.3.0. Whereas the lipophilicity (log Po/w) of the screened compounds was first computed through XLOGP3, WLOGP, MLOGP, SILICOS-IT, and iLOG predictive models. Thereafter, the actual log Po/w of a compound was calculated as an arithmetic mean of all the proposed values. Furthermore, to evaluate the bioavailability of the tested compounds, a rule-based filter employing Lipinski’s rule of five was used [55]. While the compounds with 0 or 1 violation of the described rule were considered viable to be oral drug candidates, analysis of GI absorption and BBB permeation was also made using the BOILED-Egg model [56].

#### 2.3.7. Toxicity Prediction Study

The screened phytocompounds were further evaluated for Toxicity and Lethal Dose (LD_50_) estimation using the web-based server, ProTox-II (https://tox-new.charite.de/protox_II/). The analysis was performed based on the chemical similarity between the molecular fragments of the tested compounds with those from known toxic agents. Analysis was also conducted to estimate the carcinogenicity of each candidate, predicted by the Random Forest (RF) algorithm used by ProTox-II [57].

## 3. Results

### 3.1. Maximal Electroshock (MES)-Induced Seizure Model

The *F. religiosa* extracts PE-Fr and EA-Fr were assessed for antiepileptic potential using an MES-induced seizure model. With a 400 mg/kg dose of PE-Fr and EA-Fr extracts, there was a statistically significant reduction (*p* < 0.05) in the mean duration of hind limb extension when compared with the negative control group (normal saline 0.9%) (Table 1). 

### 3.2. Molecular Docking

Molecular docking was then undertaken using Glide to virtually screen a library of 82 compounds, predict ligand binding affinities, and rank order them according to their Glide score as an estimate of binding affinity [50,58,59]. The tabulated data for the binding parameters of 7 hit compounds from *F. religiosa*, out of the screened library of 82 possibilities, is shown in Table 2. The docking scores of the top hits ranged from −6.555 kcal/mol to −13.476 kcal/mol, comparable to or higher than phenytoin used as a reference compound, which had a docking score of −6.660 kcal/mol. The binding affinities of luteolin 7-O-rutinoside (1), pelargonidin-3-rhamnoside (2), 6-C-glucosyl-8-C-arabinosyl apigenin (3), leucocyanidin (4), myricetin (5), serotonin (6) and kaempferol-3-O-rutinoside (7) were −13.476, −8.894, −8.147, −7.523, −7.171, −6.963, and −6.555 kcal/mol, respectively. The binding parameters for the other docked compounds (ligands 8 to 68 in descending order of binding affinity) have been provided as Supplementary Data (Table S1) as have their molecular structures (Supplementary Figure S1) and for those ligands with sufficient binding stability, molecular docking with Nav1.2. (Supplementary Figures S2–S37). Compounds with insufficient binding stability have not been included in the Supplementary Data.

**Figure 2:**
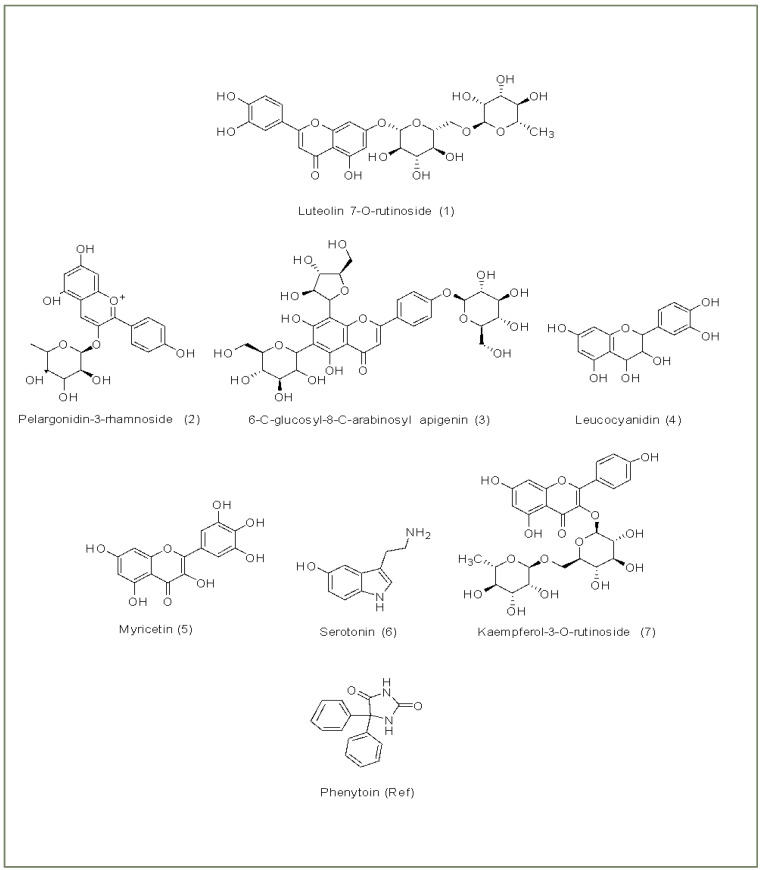
Structures of the seven hit phyto-ligands and reference compound phenytoin.

The structures of the hit compounds 1–7 are shown in Figure 2.

**Figure 3:**
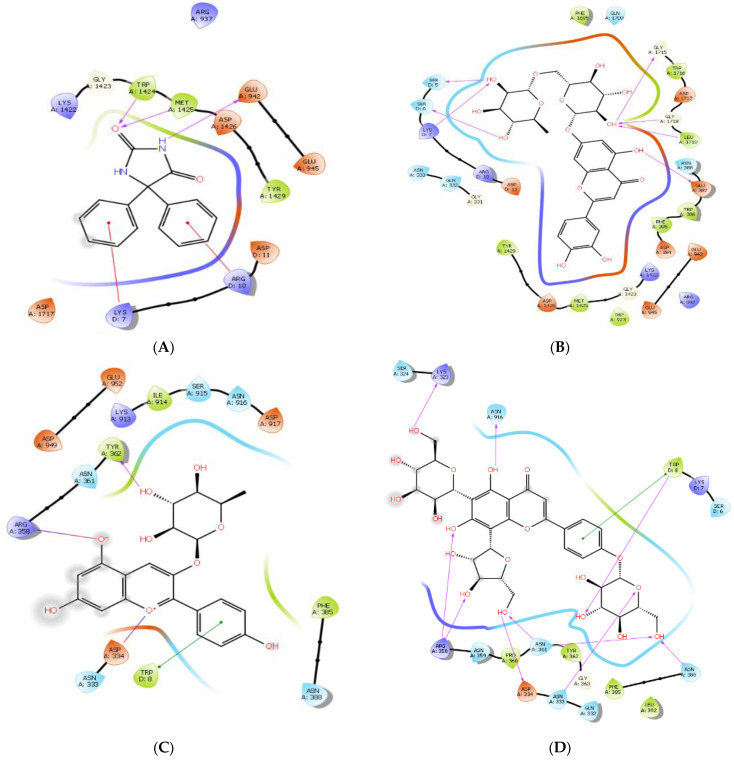

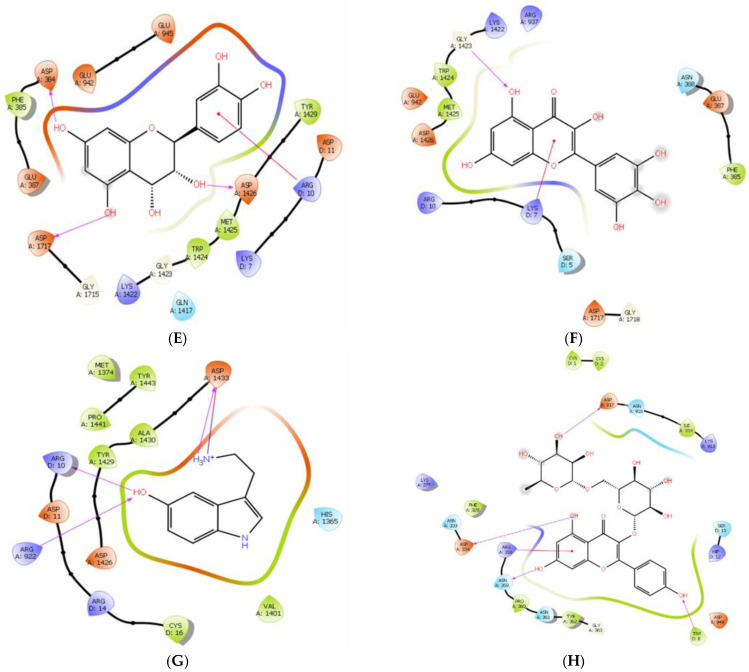
Two-dimensional interaction map analyses of the top hits for ligands screened after molecular docking as extracted from Maestro. (**A**) phenytoin, (**B**) luteolin 7-O-rutinoside, (**C**) pelargonidin-3-rhamnoside, (**D**) glucosyl-8-C-arabinosyl apigenin, (**E**) leucocyanidin, (**F**) myricetin, (**G**) serotonin, (**H**) kaempferol-3-O-rutinoside.

Post-docking diagrammatic analysis of each selected ligand was performed using PyMOL 2.5.2, and Maestro 2D interaction analyzer. These analytical tools displayed interaction patterns between the ligands and the binding pocket-forming residues of Nav1.2. As a general bifurcation, the interactions are described as either polar or non-polar and these have been presented in Table 3. To study the specific binding pattern in detail, Maestro 2D interaction map analysis was also performed to visualize hydrogen bonding, hydrophobic contacts, salt bridge formation, Van der Waals, and π–π interactions that govern the molecular recognition and shape complementarity of the docked complex in question [60], as shown in Figure 3.

**Figure 4:**
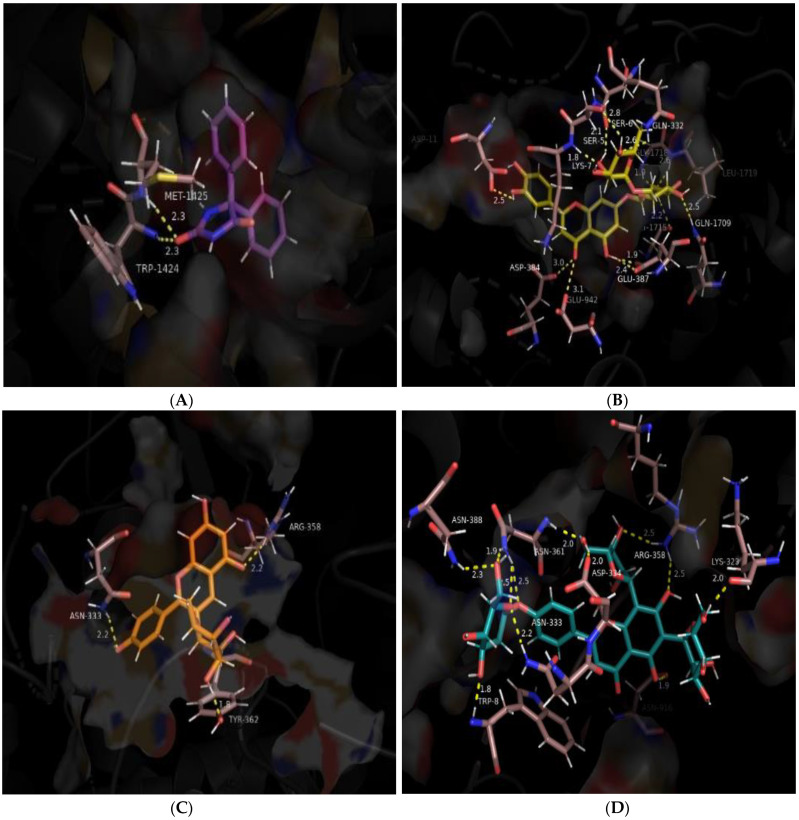

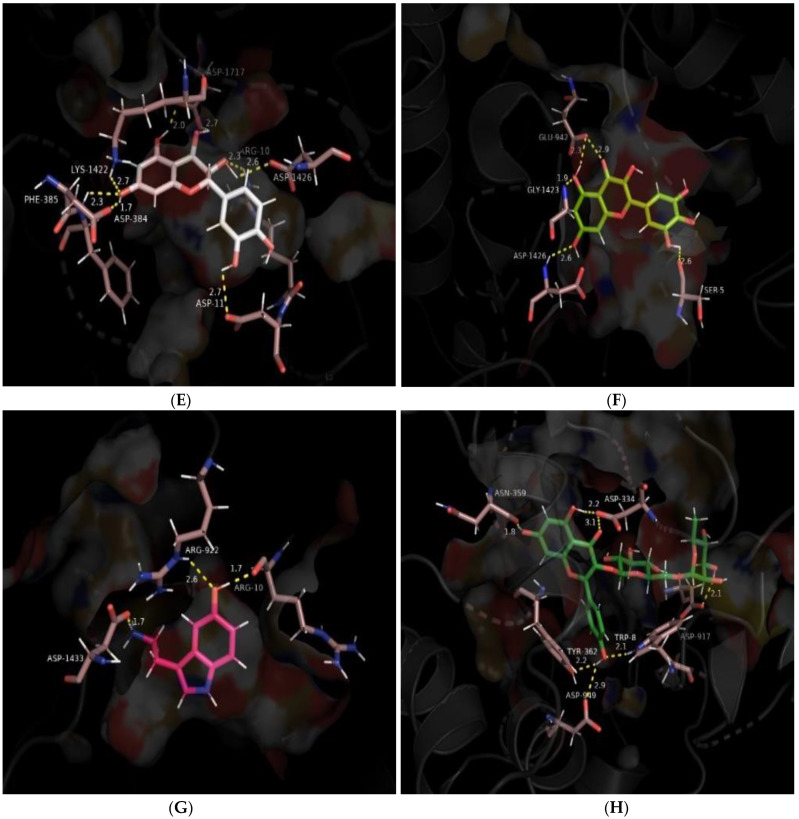
Docked poses of the phyto-ligands with human sodium channel Nav1.2 (PDB ID: 6J8E). (**A**) phenytoin (purple), (**B**) luteolin 7-O-rutinoside (olive green), (**C**) pelargonidin 3-rhamnoside (orange), (**D**) 6-C-glucosyl-8-C-arabinosyl apigenin (blue), (**E**) leucocyanidin (white), (**F**) myricetin (lime green), (**G**) serotonin (pink), (**H**) kaempferol-3-O-rutinoside (dark green). The binding pocket forming polar residues are shown in a stick representation (colored light pink) and the non-polar residues are shown as a surface (deep orange) representation with the remaining Nav1.2 shown as a cartoon (in greyscale). Hydrogen bond interactions are shown as yellow dotted lines.

Phenytoin, the reference ligand, forms hydrogen bonds with residues Met-1425 and Trp-1424 of 2.3 Ǻ each and another with the nucleophilic Glu-942 with a bond distance of 1.49 Ǻ. It also forms an aromatic hydrogen bond with Lys-7 and two π-cation interactions with Lys-7 and Arg-10 as shown in Figure 4A. The multiplicity of these contributing interactions results in a docking score of −6.660 kcal/mol.

Luteolin 7-O-rutinoside (1) possesses the highest negative (most potent) docking score of −13.476 kcal/mol. It forms seven hydrogen bonds with three consecutive residues: Ser-5, Ser-6, and Lys-7 and others with Glu-387, Gly-1715, Gly-1718, and Leu-1719. It also forms three aromatic hydrogen bonds with Glu-387, Asp-1717, and Asp-1426. The ligand forms a highly stabilizing π-sigma bond with Lys-7 and the highest number of hydrophobic bonds as shown in Table 4 and Figure 4B. Other studies have postulated that the best structural parameter correlating with binding affinity is the number of hydrophobic bonds buried after ligand binding with the protein [61], and this would provide a basis for the relatively high binding energy of luteolin 7-O-rutinoside.

The compound with the second highest binding affinity is pelargonidin-3-rhamnoside (2). Despite forming a single hydrogen bond with Tyr-362 of 1.79 Ǻ bond distance, this compound still shows an excellent docking score because it forms two highly stabilizing salt bridges with protein residues at Asp-334 and Arg-358 of 3.98 Ǻ and 3.07 Ǻ, respectively. The salt bridge with the N-atom of Arg-358 is a hydrogen-bonded salt bridge that markedly increases bridge energy. The other salt bridge between the Asp-334 residue of the protein and O-atom of the ligand, although not hydrogen bonded, is surrounded by the non-polar residues at Gln-332 and Ala-335 and is embedded in the protein core facilitating stronger electrostatic interactions [62]. Adding further to the binding energy, the ligand forms a π-π stacking with Trp-8, π-lone pair interactions with Asn-333, and π-π stacking with Tyr-362. These π–π interactions may also contribute to overall binding energies for stabilizing the ligand–protein complex contributing to the relatively high docking score of −8.894 kcal/mol, as shown in Figure 4C.

The next strongest interaction was between the target protein and 6-C-glucosyl-8-C-arabinosyl apigenin (3). The ligand docking score was −8.147 kcal/mol, which can be attributed to the presence of 10 conventional hydrogen bonds which are among the strongest interactions to stabilize a protein–ligand complex [63]. The ligand forms a hydrogen bond with Trp-8, Lys-323, Asn-333, Asp-334, Asn-388, and Asn-916, and two hydrogen bonds with Arg-358 and Asn-361, as shown in Figure 4D. In addition to forming a number of hydrogen bonds, the ligand also forms a π-π stacking with Trp-8 of 4.96 Ǻ, hydrophobic contacts with Pro-360, Tyr-362, Phe-385, Leu-392, and Van der Waals interactions with Lys-7, Ser-324, Gln-332, Asn-359, and Gly-363; all collectively contributing to the stability of the docked complex.

Leucocyanidin (4) has a binding energy of −7.523 kcal/mol attributed to cumulative strong binding from conventional hydrogen bonds formed with Asp-384 and Asp-1717 and aromatic hydrogen bonds with Glu-387 and Asp-1426. Aromatic hydrogen bonds are approximately half the strength of conventional hydrogen bonds, but they contribute to stabilizing molecular associations [64]. Leucocyanidin also forms π-cation interactions with Arg-10 of 6.03 Ǻ and π-alkyl bonding with Lys-7, as well as a number of hydrophobic and Van der Walls interactions, as shown in Table 4 and Figure 4E.

Myricetin (5); Figure 4F and serotonin (6); Figure 4G show comparable binding affinities of −7.171 kcal/mol and −6.963 kcal/mol, respectively. Myricetin forms both conventional and aromatic hydrogen bonds with residues Gly-1423 and Asp-1717, respectively, and a π-cation interaction with Lys-7 of 5.50 Ǻ resulting in a moderate docking score of −7.171 kcal/mol. Whereas, serotonin forms two aromatic hydrogen bonds with Asp-1426 and Arg-10 and two conventional hydrogen bonds with Arg-922 and Arg-10. It also forms a salt bridge of bond distance 2.74 Ǻ with Asp-1433 as seen in Figure 3. Both compounds are also stabilized through the presence of other hydrophobic and Van der Waals interactions, as detailed in Table 4.

Lastly, kaempferol-3-O-rutinoside (7) in Figure 4H shows the least docking score of −6.555 kcal/mol. It forms three conventional hydrogen bonds with Asp-334, Asn-359, and Asp-917 while forming aromatic hydrogen bonds with Asn-359 and Asn-361. A π-cation interaction is observed with protein residue Arg-358 with a 5.14 Ǻ bond distance. There are also hydrophobic interactions with Cys-1, Cys-2, Trp-8, Phe-328, Pro-360, Tyr-362, Ile-914, and Asp-949, and Van der Waals interactions with His-12, Ile-914, and Asn-916. The combination of these interactions results in the binding energy score.

**Figure 5:**
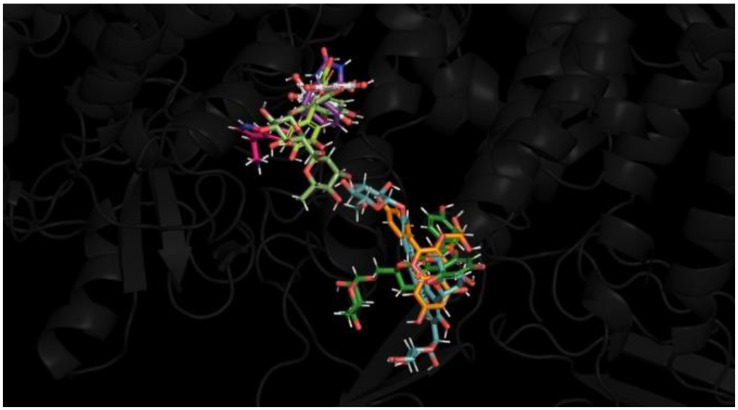
Modeling of the best-docked ligands at the active site region of human Nav1.2. Luteolin 7-O-rutinoside (olive green), pelargonidin-3-rhamnoside (orange), 6-C-glucosyl-8-C-arabinosyl apigenin (blue), leucocyanidin (white), myricetin (lime green), serotonin (pink), kaempferol-3-O-rutinoside (dark green) and phenytoin. The reference molecule is tagged in purple, with all molecules superimposed at the active site region of Nav1.2 channel using Pymol 2.5.2.

The orientation of the best-docked ligands at the active site region of the human Nav1.2 channel is shown in Figure 5.

### 3.3. MM/GBSA Studies

The docked ligands were further evaluated by calculating the binding free energy (∆Gbind) of protein–ligand complexes using Prime MM/GBSA studies [65]. This software utilizes molecular mechanics (force fields) and continuum (implicit) solvation models [60] to calculate the relative free energy of two end-states of each ligand molecule [66]. The results in Table 1 indicate that the ΔGbind values of all selected ligands fell within the range of −21.72 kcal/mol (myricetin) to −64.73 kcal/mol (kaempferol-3-O-rutinoside), with kaempferol-3-O-rutinoside (7), luteolin 7-O-rutinoside (1) and 6-C-glucosyl-8-C-arabinosyl apigenin (3) producing ΔGbind values of −64.73 kcal/mol, −62.13 kcal/mol, and −46.82 kcal/mol, respectively. Whereas pelargonidin-3-rhamnoside (2), serotonin (6) leucocyanidin (4), and myricetin (5) showed relative binding affinity values of −45.82 kcal/mol, −41.45 kcal/mol, −24.34 kcal/mol, and −21.72 kcal/mol, respectively. Since MM/GBSA binding energies are approximate free energies of binding, a more negative value signifies a stronger binding [67]. Therefore, this study suggests that kaempferol-3-O-rutinoside (7), luteolin 7-O-rutinoside (1), and 6-C-glucosyl-8-C-arabinosyl apigenin (3) display the best three theoretical free energy binding values.

### 3.4. Molecular Dynamics Simulation

**Figure 6:**
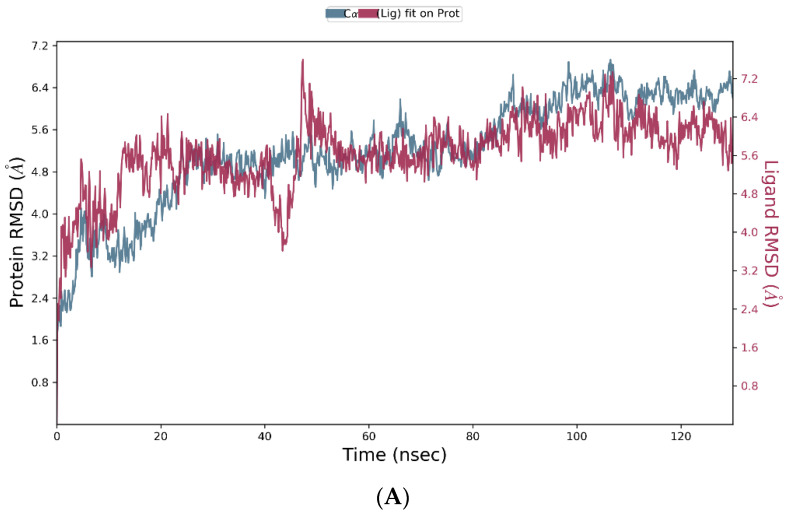

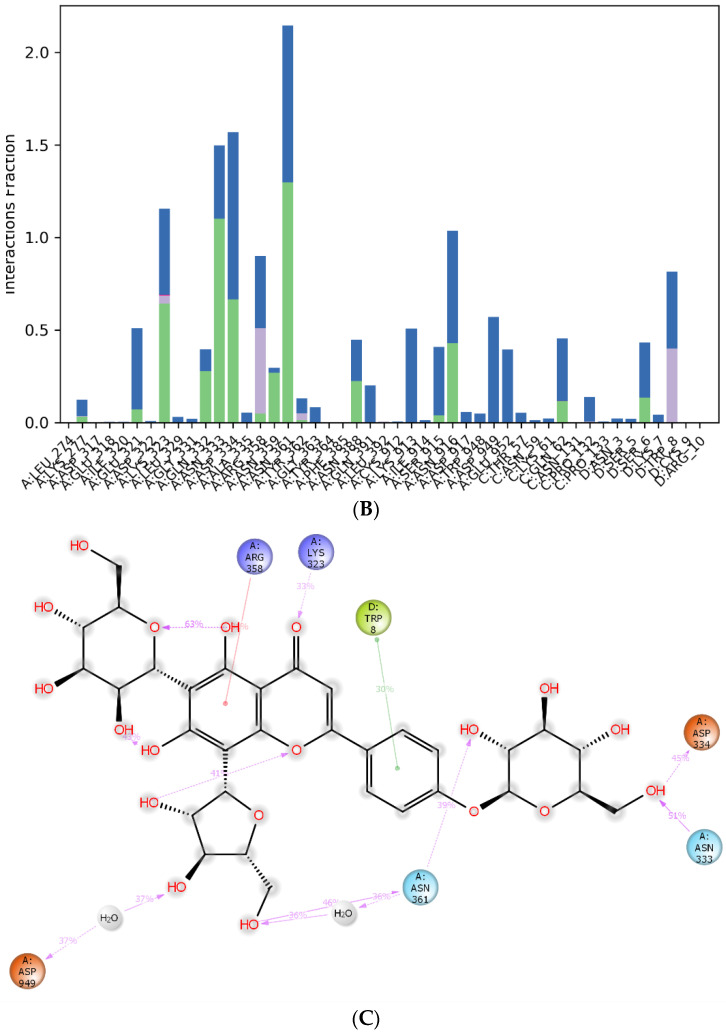
MD simulation studies of 6-C-glucosyl-8-C-arabinosyl apigenin in complex with Nav1.2. (**A**) RMSD plot of protein backbone (Cα) and protein conformational change during ligand binding. (**B**) Interaction fraction plot showing different Nav1.2 residues that interact with the ligand during a 130 ns MD simulation. (**C**) Interaction of ligand atoms with the Nav1.2 residues that occur for more than 30% of the simulation time.

**Figure 7:**
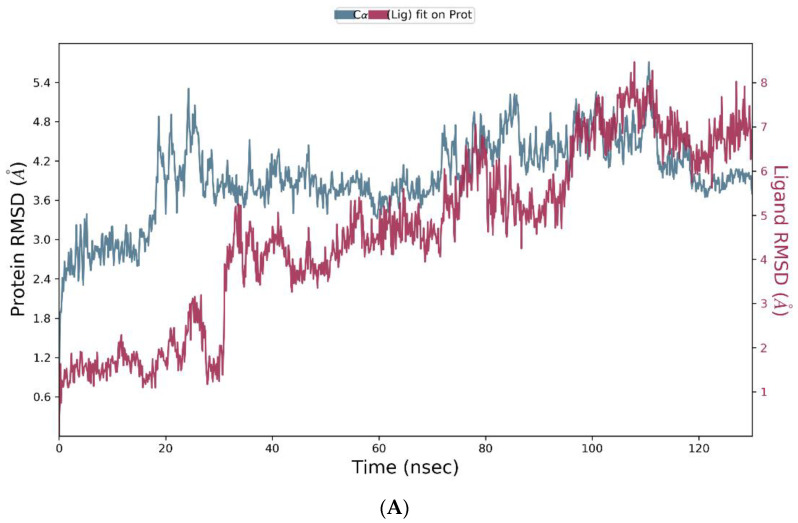

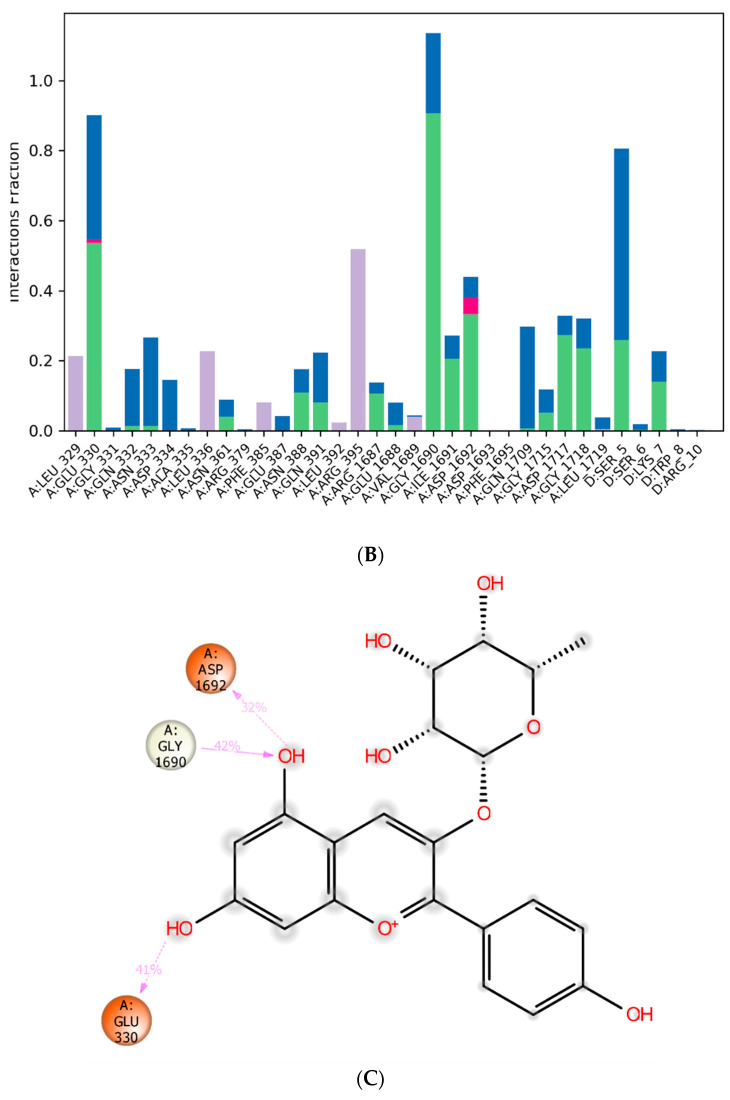
Simulation studies of pelargonidin-3-rhamnoside in complex Nav1.2. (**A**) RMSD plot of protein backbone (Cα) and protein conformational change during ligand binding. (**B**) Interaction fraction plot showing different protein residues that interact with the ligand during a 130 ns MD simulation. (**C**) Interaction of ligand atoms with the protein residues that occur for more than 30% of the simulation time.

The Nav1.2-ligand complexes with the highest docking scores (Nav1.2-luteolin 7-O-rutinoside, Nav1.2-pelargonidin-3-rhamnoside, Nav1.2-6-C-glucosyl-8-C-arabinosyl apigenin, and Nav1.2-leucocyanidin) were subjected to molecular dynamics (MD) simulations at NPT for 130 ns. The subsequent MD trajectory analysis explored the binding stability of the heterodimer-Nav1.2 complexes using the root mean square deviation (RMSD) fingerprints and protein–ligand (P–L) interaction mapping. The RMSD plots measured the conformational changes in the Nav1.2 backbone when bound to the respective ligand in comparison to its initial spatial conformation [68], and intermolecular contacts of Nav1.2 and the bound ligands were recorded as interaction fraction mapping. Of all the simulated complexes, 6-C-glucosyl-8-C-arabinosyl apigenin (3) and pelargonidin-3-rhamnoside (2) formed stable complexes as shown in Figure 6 and Figure 7, respectively, whereas the simulation results of the remaining compounds (luteolin 7-O-rutinoside and leucocyanidin) have been included in the Supplementary Data section (Supplementary Figures S38 and S39, respectively).

The RMSD plot for 6-C-glucosyl-8-C-arabinosyl apigenin and Nav.12, as shown in Figure 6A, indicates the complex to display optimum stability with some inherent fluctuations between 10 and 25 ns, 40 and 50 ns, and then at 110 and 130 ns. Figure 6B indicates the interaction fraction plot of the bound complex, in which Asn-361 forms 100% stable hydrogen bonds and water bridges. Asn-333, Asp-334, and Asn-916 also form 100% stable hydrogen bonds and water bridges, Lys-323 and Arg-358 also form stabilized hydrogen bonds, hydrophobic contacts, and water bridges. Furthermore, Ser-6, Trp-8, Gln-62, Asp-949, Glu-321, Gln-332, Asn-388, Lys-913, and Glu-952 are some of the other protein residues responsible for stabilizing the protein–ligand complex during 40–80% of the simulation time. Figure 6C indicates a detailed assessment of interacting residues in which 6-C-glucosyl-8-C-arabinosyl apigenin forms a 63% stable π-cation interaction with Arg-358, 51% and 45% stable hydrogen bonding with Asn-333 and Asp-334, respectively. While the ligand forms 30% stable π-π stacking with Trp-8, it also forms 37%, 36%, and 33% stable hydrogen bonds with Asp-949, Asn-361, and Lys-323, respectively, for more than 30% of the simulation time.

An initial MD simulation of the pelargonidin-3-rhamnoside-Nav1.2 complex for 130 ns did not show configurational complementarity but eventually stabilized between 58 and 78 ns and 92 and 122 ns as shown in Figure 7A. The interaction plot of Nav1.2 with pelargonidin-3-rhamnoside, as shown in Figure 7B, indicates that Gly-1690 forms hydrogen bonds and water bridges for 100% of the simulation time. In addition, Glu-330 forms hydrogen bonds, water bridges, and ionic contacts with the ligand for 90% of the simulation time, and Ser-5 forms hydrogen bonds and water bridges for 80% of the simulation time. Leu-329, Leu-336, Phe-385, Leu-392, Arg-395, and Val-1689 form highly stabilizing hydrophobic contacts. Lys-7, Gln-332, Asn-333, Asp-334, Asn-388, Gln-391, Ile-1691, Gln-1709, Gly-1715, Asp-1717, and Gly-1718 are other interacting protein residues that contribute to the formation of stable hydrogen bonds and water bridges within the protein–ligand complex. Figure 7C summarizes the interacting residues in which Gly-1690, Glu-330, and Asp-1692 form 42%, 41%, and 32% stable hydrogen bonds, respectively.

### 3.5. Drug-likeness Predictions

The results of the physiochemical and pharmacokinetic analysis performed using SwissADME and ADMET-SAR web servers have been presented in Table 5. Regarding the physiochemical properties, the tabulated data suggests that pelargonidin-3-rhamnoside (2), leucocyanidin (4), myricetin (5), and serotonin (6) hold optimal drug-like characters with only one or no Lipinski’s violations.

Luteolin 7-O-rutinoside (1), kaempferol-3-O-rutinoside (7), and 6-C-glucosyl-8-C-arabinosyl apigenin (3) violate Lipinski’s rule of five since each structure has three potential violations rather than a maximum of one violation for a promising drug candidate taken orally [55]. Each of these structures possesses greater than acceptable H-bond acceptors and donors with a molecular weight exceeding the Lipinski recommended range of <500g/mol having values of 610.52 g/mol, 722.69 g/mol, and 594.52 g/mol, respectively.

While leucocyanidin (4) and serotonin (6) show high GI absorption, all other screened phytochemicals have predicted low GI absorption profiles, but not in the very low to no GI absorption classes. However, a limitation is that only serotonin (6) has predictive capability for blood–brain barrier (BBB) penetrance.

### 3.6. Toxicity Predictions

The ProTox-II analysis of the docking hits is summarized in Table 6 and revealed that six of seven compounds are relatively non-toxic, with myricetin (5) potentially toxic if consumed, and all compounds were non-carcinogenic.

## 4. Discussion

In this study, we examined whether *F. religiosa* contained phytochemicals capable of acting as anticonvulsants using an in vivo MES model, which induces tonic–clonic convulsions in mice. Pre-treatment of mice with the *F. religiosa* test extracts (PE-Fr and EA-Fr) showed an amelioration of the tonic hind limb extensor (THLE) phase of the induced convulsions, indicating the potential of *F. religiosa* constituents to be biologically active. Furthermore, this demonstrated the ability of *F. religiosa* phytochemicals to cross the blood–brain barrier, a requirement for efficacy in treating grand mal epilepsy. Other studies using extracts from *F. religiosa* have also confirmed its potential to contain antiepileptic agents through pharmacological evaluations [24,69]. The fruit extract of *F. religiosa* modulates serotonergic pathways [24], the bark extract is effective against GABAergic epileptic pathways [25], and saponins from the adventitious roots of *F. religiosa* produce an anticonvulsive effect through the modulation of GABAergic, Na^+^ and Ca^2+^ channels [70]. Our study further extends the work in this area by considering a possible mechanism of action of the phytochemical anticonvulsants via their targeted binding to the Nav1.2 channel. An in silico strategy was used to characterize the known compounds from *F. religiosa* for their potential binding to the Nav1.2 channel. Out of the 82 computationally screened *F. religiosa* phytocompounds, seven exhibited better predicted Nav1.2 binding potential than phenytoin, a drug established for its antiepileptic effects [42]. 

The computational techniques that were employed included molecular docking studies, prime MM/GBSA analysis, and MD simulations to predict the interactions, binding energetics, and stability of possible ligand compounds bound to the μ-conotoxin KIIIA Nav1.2 target molecule, respectively. The Nav α-subunits have at least six binding sites for neurotoxins, and these have been classified as neurotoxin receptor sites 1–6 [71]. The μ-conotoxin KIIIA ligand used as the basis for structural studies binds to Site 1, as does tetrodotoxin and saxitoxin [6,71]. These neurotoxins inhibit VGSC activity by occlusion of the channel pore and thereby block Na^+^ conductance [6,71]. Phenytoin, the reference drug used in our study, also binds extracellularly at domains II-S6 and III-S6, occluding the channel pore [72,73]. Hence, our study considered phyto-ligands with potential for high affinity binding to Site 1 but did not consider their potential binding to other neurotoxin receptor sites.

Seven compounds with notable docking scores were selected after computational screening for further assessment through MM/GBSA and MD simulation studies, implementing a funnel approach to refine potential drug candidates. Amongst these compounds, pelargonidin-3-rhamnoside (2) and 6-C-glucosyl-8-C-arabinosyl apigenin (3) demonstrated favorable dynamic stability with Nav1.2 in MD simulations. These compounds interacted with Trp-8, His-12, Gln-332, Asn-333, Asn-361, and Phe-385 residues of the Nav1.2 binding site.

Pelargonidin-3-rhamnoside showed a satisfactory binding profile with Nav1.2. The potential for its antiepileptic activity has not previously been reported in the literature. However, pelargonidin-3-rhamnoside is an anthocyanidin glycoside, and anthocyanins have reputed neuroprotective activities [74,75,76]. Thus, pelargonidin-3-rhamnoside represents an attractive candidate for further research as an antiepileptic.

Similarly, our study also identified the Nav1.2 blocking potential of 6-C-glucosyl-8-C-arabinosyl apigenin; a C-glycosyl flavonoid derivative found in whole-wheat spaghetti [77]. The neurovascular protective effects of apigenins have been cited to combat various psychiatric and neurological disorders including anxiety, depression, Parkinson’s, and Alzheimer’s disease [78], but the use of 6-C-glucosyl-8-C-arabinosyl apigenin as a treatment for epilepsy will be important to explore in future research. 

Luteolin-7-O-rutinoside (1) is a plant polyphenolic secondary metabolite that is a flavonoid (C6-C3-C6 structure) with antioxidant, anti-inflammatory, and free radical scavenger abilities [79,80]. Luteolin, a chemical analog of luteolin-7-O-rutinoside, has been evaluated in an MES test and was not an effective anti-convulsant [81] but whether luteolin-7-O-rutinoside is an effective anti-epileptic has yet to be considered. Similarly, for the flavonoid, leucocyanidin, there are no studies that have considered its ability to act as an anti-epileptic. Myricetin, another natural flavonoid, antioxidant, and nutraceutical, has a range of reported pharmacological effects; including those that are anti-inflammatory, anti-tumor, and anti-diabetic [82]. Myricetin administration was able to reduce seizure and mortality rates in a pentylenetetrazole(PTZ)-induced mouse model of epilepsy, and this was attributed to its ability to regulate the BDNF-TrkB signaling pathway and modulate matrix metalloproteinase-9 and GABA_A_ receptor expression [83]. Serotonin (5-hydroxytryptamine) (5-HT) is a monoamine with a plethora of biological functions including activity as a neurotransmitter through binding to serotonin (5-HT) receptors. Activity of the serotonergic system has been implicated in multiple aspects of epilepsy [84], however, binding to Nav1.2 and modulation of epileptogenesis has not yet been considered. Kaempferol-3-O-rutinoside is a flavonol glycoside with antioxidant, anti-inflammatory, and hepatoprotective activities [85]. Kaempferol-3-O-rutinoside has no reported anti-epileptic activity but its parent analog, kaempferol, was able to delay seizures in a PTZ-induced epilepsy model [86].

The applied in silico techniques provided insight into the binding modes of ligand–protein macromolecular complexes and pharmacokinetics and physiochemical properties were also considered using predictive approaches. Through the use of the comprehensive SwissADME, and ProTox-II databases, several of the proposed antiepileptic compounds displayed useful ADMET profiles. Furthermore, only myricetin was predicted to be potentially toxic (Table 6), but this is in contrast to its commercial availability and listing as non-hazardous [87]. Similarly, luteolin-7-O-rutinoside, leucocyanidin, and kaempferol-3-O-rutinoside are commercially available and are all listed as non-hazardous chemicals [88,89,90]. Serotonin, as an endogenous monoamine is relatively non-toxic: acute toxicity of oral serotonin hydrochloride has an LD_50_ of 60 mg/kg in mice [91]. There is no toxicity data for pelargonidin-3-rhamnoside and 6-C-glucosyl-8-C-arabinosyl apigenin, but presumably, they follow the prediction of relatively non-toxic.

## 5. Conclusions

*F. religiosa* contains phytochemicals that can act as anticonvulsants and could provide compounds for novel antiepileptic drugs through binding and inhibition of the activity of the Nav1.2. voltage-gated sodium channel. Furthermore, these compounds could provide the building blocks for further structural and chemical derivatization that may improve binding as well as pharmacokinetics and could be manipulated to form prodrugs to facilitate chemical delivery and improve drug efficacy. However, a limitation of the current study is that additional experimental validation of individual phytocompounds and possible alternative epileptic models will be needed to fully assess the specific antiepileptic properties and modes of action of these phytochemicals prior to any consideration of their safety and efficacy in humans. Furthermore, although we have undertaken toxicity predictions, the potential toxicity of the phytocompounds will also need to be evaluated in vivo.

## Figures and Tables

**Table 1:** Maximal electroshock-induced seizure model responses to phenytoin and F. religiosa extracts.

Group	Treatment Groups	Pre-Treatment	No. of Animals	Onset Time for THLE (sec)
1	Control	Normal Saline	6	11.00 ± 2.97
2	Standard	Phenytoin	6	1.17 ± 1.33 **
3	PE-Fr	PE-Fr	6	2.67 ± 1.96 *
4	EA-Fr	EA-Fr	6	3.00 ± 1.26 *

Onset time values are means ± S.D. Statistical evaluation was performed using a one-way ANOVA followed by Dunn’s multiple comparison test. For marked significance: ** *p* < 0.01, * *p* < 0.05 when compared with the control (normal saline) treatment. Abbreviations: PE-Fr, petroleum ether fraction of *Ficus religiosa*; EA-Fr, ethyl acetate fraction of *Ficus religiosa*; THLE, tonic hind limb extension.

**Table 2:** Molecular docking and Prime MM/GBSA results of screened phyto-ligands with the target protein Nav1.2.

							Prime MM/GBSA
Ligand	Name	Molecular Formula	Molecular Weight (g/mol)	Docking Score	XPGScore	Glide Score	ΔG_bind_
			g/mol	kcal/mol	kcal/mol	kcal/mol	kcal/mol
1	Luteolin 7-O-rutinoside	C_27_H_30_O_15_	610.5	−13.476	−13.476	−13.476	−62.13
2	Pelargonidin-3- rhamnoside	C_21_H_21_O_9_+	417.4	−8.894	−10.301	−10.301	−45.82
3	6-C-glucosyl-8-C- arabinosyl apigenin	C_26_H_28_O_14_	726.2	−8.147	−8.191	−8.191	−46.82
4	Leucocyanidin	C_15_H_14_O_7_	306.3	−7.523	−7.523	−7.523	−24.34
5	Myricetin	C_15_H_10_O_8_	318.2	−7.171	−7.209	−7.209	−21.72
6	Serotonin	C_10_H_12_N_2_O	176.2	−6.963	−6.963	−6.963	−41.45
7	Kaempferol-3-O- rutinoside	C_27_H_30_O_15_	594.5	−6.555	−6.584	−6.584	−64.73
Ref	Phenytoin	C_15_H_12_N_2_O_2_	252.3	−6.660	−7.022	−7.022	−25.80

**Table 3:** Polar and non-polar interacting residues with ligands in the Nav1.2 binding pocket.

	Interaction Type	Interacting Residues
Phenytoin	Polar Non-polar	Glu-942, Trp-1424, Met-1425 Ser-5, Lys-7, Trp-8, Arg-10, Asp-11, His-12, Trp-923, Arg-937, Glu-945, Phe-1421, Lys-1422, Gly-1423, Asp-1426, Tyr-1429, Gly-1715, Asp-1717, Gly-1718
Luteolin 7-O- rutinoside	Polar Non-polar	Ser-5, Ser-6, Lys-7, Asp-11, Glu-387, Gln-1709, Gly-1715, Gly-1718, Leu-1719 Trp-8, Arg-10, His-12, Gly-331, Asn-333, Asn-361, Asp-384, Phe-385, Trp-386, Asn-388, Gln-391, Trp-923, Arg-937, Glu-942, Glu-945, Lys-1422, Gly-1423, Met-1425, Asp-1426, Tyr-1429, Gly-1690, Phe-1695, Trp-1716, Asp-1717, Leu-1720
Pelargonidin-3-rhamnoside	Polar Non-polar	Asn-333, Arg-358, Tyr-362 Ser-6, Trp-8, His-12, Lys-323, Gln-332, Asp-334, Ala-335, Asn-361, Tyr-364, Phe-385, Asn-388, Lys-913, Ile-914, Ser-915, Asn-916, Asp-917, Asp-949, Glu-952
6-C-glucosyl-8-C-arabinosyl apigenin	Polar Non-polar	Trp-8, Lys-323, Asn-333, Asp-334, Arg-358, Asn-361, Asn-388, Asn-916 Ser-5, Ser-6, Lys-7, Cys-9, Lys-277, Asp-322, Ser-324, His-325, Phe-326, Gly-331, Gln-332, Leu-336, Asn-359, Pro-360, Tyr-362, Gly-363, Arg-379, Phe-385, Leu-392, Ser-915, Asp-917, Glu-945, Trp-948
Leucocyanidin	Polar Non-polar	Arg-10, Asp-11, Asp-384, Phe-385, Lys-1422, Asp-1426, Asp-1717 Lys-7, Trp-8, His-12, Trp-386, Glu-387, Trp-923, Arg-937, Glu-942, Trp-943, Glu-945, Gln-1417, Gly-1423, Trp-1424, Met-1425, Tyr-1429, Ala-1714, Gly-1715, Gly-1718
Myricetin	Polar Non-polar	Ser-5, Glu-942, Gly-1423, Asp-1426 Ser-6, Lys-7, Arg-10, Phe-385, Glu-387, Asn-388, Arg-937, Phe-1421, Lys-1422, Trp-1424, Met-1425, Tyr-1429, Gly-1715, Asp-1717, Gly-1718, Ala-1721
Serotonin	Polar Non-polar	Arg-10, Arg-922, Asp-1433 Asp-11, Ser-13, Arg-14, Cys-15, Cys-16, Phe-1363, His-1365, Met-1374, Val-1401, Met-1425, Asp-1426, Ile-1427, Tyr-1429, Ala-1430, Pro-1441, Lys-1442, Tyr-1443
Kaempferol-3-O- rutinoside	Polar Non-polar	Trp-8, Asp-334, Asn-359, Tyr-362, Asp-917, Asp-949 Cys-1, Cys-2, Cys-9, His-12, Ser-13, Lys-277, Phe-328, Asn-333, Ala-335, Arg-358, Pro-360, Asn-361, Gly-363, Tyr-364, Ile-914, Ser-915, Asn-916, Glu-952

**Table 4:** A summary of the interactive forces responsible for the binding of the docked ligand complexes to human Nav1.2.

Structure	Ligand	Hydrogen Bonding	Salt Bridges/(π-π)/(π-Cation)/π-Sigma Interactions	Other Hydrophobic Contacts	Van der Waals Interactions
Ref	Phenytoin	Lys-7, Glu-942, Trp-1424, Met-1425, Asp-1426	Arg-10 (π-cation)	Tyr-1429	Asp-11, Glu-945, Lys-1422, Gly-1423, Asp-1426, Asp-1717
1	Luteolin 7-O-Rutinoside	Ser-5, Ser-6, Lys-7, Glu-387, Gly-1715, Gly-1718, Leu-1719	Lys-7 (π-sigma)	Asp-384, Phe-385, Trp-386, Trp-923, Met-1425, Tyr-1429, Phe-1695, Gln-1709, Trp-1716	Arg-10, Gly-331, Gln-332, Asn-333, Arg-937, Glu-942, Glu-945, Gly-1423, Asp-1426, Tyr-1429
2	Pelargonidin-3-Rhamnoside	Ser-5, Glu-330, Tyr-362, Gly-1690, Asp-1692	Trp-8 (π-π), Asn-333 (π-lone pair), Asp-334 (salt bridge), Arg-358, Tyr-362 (π-sigma)	Leu-329, Leu-336, Phe-385, Leu-392, Arg-395, Ile-914, Val-1689	-
3	6-C-Glucosyl-8-C-Arabinosyl Apigenin	Trp-8, Lys-323, Asn-333, Asp-334, Arg-358, Asn-361, Asn-388, Asn-916	Trp-8 (π-π T-shaped stacking)	Pro-360, Tyr-362, Phe-385, Leu-392	Lys-7, Ser-324, Gly-331, Gln-332, Asn-359, Gly-363
4	Leucocyanidin	Asp-384, Glu-387, Asp-1426, Asp-1717	Lys-7 (π-alkyl), Arg-10 (π-cation)	Phe-385, Trp-1424, Met-1425, Tyr-1429	Gln-1417, Gly-1423
5	Myricetin	Gly-1423, Asp-1717	Lys-7 (π-cation/π -alkyl)	Phe-385, Trp-1424, Met-1425	Arg-10, Arg-937, Glu-942, Lys-1422, Gly-1718
6	Serotonin	Arg-10, Arg-922, Asp-1426	Asp-1433 (salt bridge)	Cys-16, Met-1374, Val-1401, Tyr-1429, Ala-1430, Pro-1441, Tyr-1443	Asp-11, Arg-14, His-1365
7	Kaempferol-3-O-Rutinoside	Trp-8, Asp-334, Asn-359, Asn-361, Asp-917	Arg-358 (π-cation)	Cys-1, Cys-2, Phe-328, Pro-360, Tyr-362, Ile-914, Asp-949	His-12, Ile-914, Asn-916

**Table 5:** In silico ADME analysis of the Nav1.2 ligands.

Ligand	MW (g/mol)	HB Acceptor	HB Donor	Log P	Molar Refractivity	Rule of Five Violations	Bio- Availability Score	GI Absorption	BBB Permeation
Luteolin 7-O- rutinoside	610.52	16	10	2.81	140.52	3	0.17	Low	-
Pelargonidin-3-rhamnoside	417.39	9	6	−1.61	105.11	1	0.55	Low	-
6-C-glucosyl-8-C-arabinosyl apigenin	726.23	17	11	2.16	171.97	3	0.17	Low	-
Leucocyanidin	306.27	7	6	1.19	75.50	1	0.55	High	-
Myricetin	318.24	8	6	1.08	80.06	1	0.55	Low	-
Serotonin	176.21	2	3	1.18	52.80	0	0.55	High	+
Kaempferol-3-O-rutinoside	594.52	15	9	2.79	139.36	3	0.17	Low	-

Molecular weight (recommended range: <500); hydrogen bond acceptor (recommended range: ≤10); hydrogen bond donor (recommended range: ≤5); lipophilicity/LogP (recommended range: <5); molar refractivity (recommended range: 40-130); number of violations of Lipinski’s rule, acceptable range: 1 [55].

**Table 6:** Toxicity predictions of the Nav1.2 ligands.

Structure Number	Ligand	Predicted LD_50_ (mg/kg)	Predicted Toxicity Class	Carcinogenicity
1	Luteolin-7-O-rutinoside	5000	5	-
2	Pelargonidin-3-rhamnoside	5000	5	-
3	6-C-glucosyl-8-C- arabinosyl apigenin	2300	5	-
4	Leucocyanidin	2500	5	-
5	Myricetin	159	3	-
6	Serotonin	2300	5	-
7	Kaempferol-3-O-rutinoside	5000	5	-

Class 1: deadly if consumed (LD50 ≤ 5 mg/kg); Class 2: deadly if consumed (5 < LD50 ≤ 50 mg/kg); Class 3: lethal if consumed (50 < LD50 ≤ 300 mg/kg); Class 4: harmful if consumed (300 < LD50 ≤ 2000 mg/kg); Class 5: maybe harmful if consumed (2000 < LD50 ≤ 5000 mg/kg); Class 6: non-lethal (LD50 > 5000 mg/kg) [57].

## Data Availability

The data supporting this study are available as Supplementary Data.

## Supplementary Materials

The following supporting information can be downloaded at: https://www.mdpi.com/article/10.3390/brainsci14060545/s1, Table S1. Molecular docking results for docked ligands against sodium channel Nav1.2. Figure S1. Molecular structures of the docked compounds. Figures S2–S37. Phyto-ligands in complex with sodium channel Nav1.2. Figure S38. MD simulation studies of luteolin 7-O-rutinoside in complex with Nav1.2. Figure S39. MD simulation studies of leucocyanidin in complex with Nav1.2.
