# Supplementary material for: An In Vivo and In Silico Approach Reveals Possible Sodium Channel Nav1.2 Inhibitors from Ficus religiosa as a Novel Treatment for Epilepsy
Source: Brain Sci. 2024 May 27;14(6):545. doi: 10.3390/brainsci14060545 (PMC11202011; doi:10.3390/brainsci14060545)
Supplement: Supplementary file 1 [file brainsci-14-00545-s001.zip › brainsci-3031089-supplementary.pdf]

Supplementary Data:

Supplementary Table S1. Molecular docking results for docked ligands against sodium channel $Na_v1.2$.

Ligand	Name	Docking score	GlideScore	Glide Energy
8.	Quercetin	-6.717	-6.749	-48.087
9.	Methyl piperate	-6.717	-6.749	-48.087
10.	3,4,5,7-tetrahydroxy-3-methoxyflavone	-6.206	-6.239	-38.272
11.	Inositol	-5.860	-5.860	-33.925
12.	Apigenin	-5.683	-5.723	-36.629
13.	Adipoin	-5.588	-5.588	-19.967
14.	Caffeic acid	-5.552	-5.552	-27.708
15.	Eugenol	-5.503	-5.503	-25.108
16.	leucoanthocyanidin	-5.462	-5.462	-29.881
17.	Salicylaldehyde	-5.381	-5.430	-27.875
18.	Kaempferol	-5.377	-5.409	-37.866
19.	Catechol	-5.152	-5.153	-23.760
20.	Alpha-cubebene	-4.981	-4.981	-21.780
21.	(E)-Linalool oxide	-4.926	-4.926	-27.598
22.	Benzeneacetonitrile	-4.813	-4.813	-22.463
23.	δ-cadinene	-4.732	-4.732	-24.908
24.	Phenylacetaldehyde	-4.687	-4.687	-22.269
25.	γ-cadinene	-4.657	-4.657	-21.821
26.	Ferulic acid	-4.638	-4.638	-31.041
27.	p-Vinylguaiacol	-4.531	-4.531	-26.004
28.	2-Phenylethyl alcohol	-4.529	-4.529	-24.413
29.	Bergapten	-4.441	-4.441	-25.381
30.	Benzyl alcohol	-4.365	-4.365	-23.535

31.	4H-Pyran-4-one,2,3-dihydro-3,5-dihydroxy-6-methyl	-4.300	-4.390	-23.664
32.	28-Isofucosterol	-4.290	-4.290	-29.097
33.	3-Methylcyclopenetane-1,2-dione	-4.180	-4.590	-22.776
34.	Dihydroactinidiolide	-4.171	-4.171	-20.672
35.	stigmasterol	-3.857	-3.857	-28.368
36.	(E)-Cinnamyl alcohol	-3.669	-3.669	-22.007
37.	α-amyrin	-3.621	-3.621	-35.733
38.	vitamin k1	-3.601	-3.601	-36.902
39.	β-Eudesmol	-3.461	-3.461	-27.773
40.	α-Eudesmol	-3.430	-3.430	-29.925
41.	ergost-5-en-3-ol(3beta)	-3.429	-3.429	-29.360
42.	α- thujene	-3.357	-3.357	-18.100
43.	α-Copaene-11-ol	-3.354	-3.354	-21.481
44.	α-Cadinol	-3.334	-3.334	-18.249
45.	Bergaptol	-3.325	-3.519	-24.174
46.	limonene	-3.247	-3.247	-21.071
47.	(E)-β-Ionone	-3.239	-3.239	-21.318
48.	Itaconic anhydride	-3.229	-3.229	-18.707
49.	Lanosterol	-3.203	-3.203	-28.871
50.	Piper longumine	-3.180	-3.180	-40.371
51.	β-sitosterol	-3.151	-3.151	-15.639
52.	γ-Eudesmol	-3.125	-3.125	-29.628
53.	P-coumaric acid	-3.123	-3.123	-24.555
54.	lanosta-8,24-dien-3-ol, acetate (3 beta)	-3.094	-3.094	-41.764
55.	α-bergamotene	-2.916	-2.916	-21.944
56.	α-ylangene	-2.681	-2.681	-15.280

57.	Cholest-5-en-3-ol (3 beta)	-2.679	-2.679	-2.284
58.	α-copaene	-2.677	-2.677	-18.395
59.	Germacrene-D	-2.575	-2.575	-17.497
60.	Camphene	-2.496	-2.496	-13.707
61.	aromadendrene	-2.473	-2.473	-15.180
62.	Caffeine	-2.414	-2.414	-25.485
63.	β-amyrin	-2.338	-2.338	-29.061
64.	bicyclogermacrene	-2.046	-2.046	-24.110
65.	alloaromadendrene	-1.961	-1.961	-21.404
66.	Urs-12-en-24-oic acid, 3-oxo-methyl ester	-1.937	-1.937	-35.459
67.	(3Z)-Hexenyl benzoate	-1.902	-1.902	-21.977
68.	9,19-Cyclolanost_24-en-3-ol (3 beta	-1.893	-1.893	-27.648

Supplementary Figure S1. Molecular structures of the docked compounds.

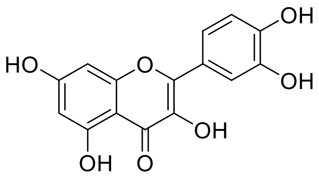

Quercetin (8)

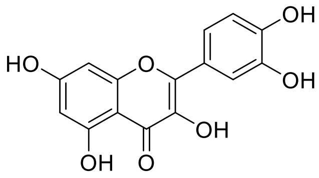

Methyl piperate (9)

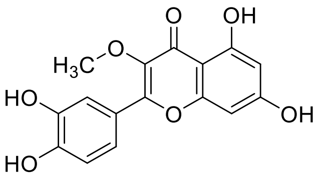

3,4,5,7-tetrahydroxy-3-methoxyflavone (10)

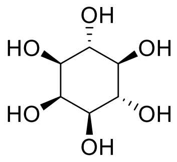

Inositol (11)

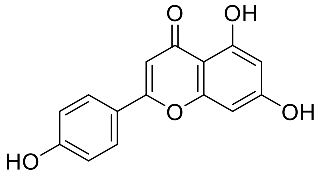

Apigenin (12)

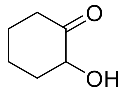

Adipoin (13)

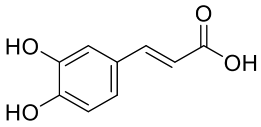

Caffeic acid (14)

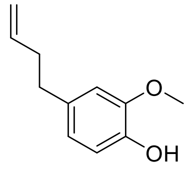

Eugenol (15)

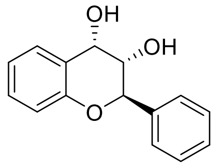

leucoanthocyanidin (16)

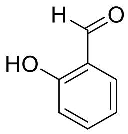

Salicylaldehyde (17)

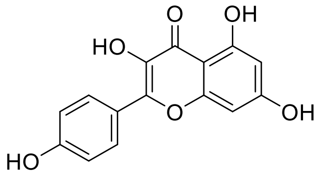

Kaempeferol (18)

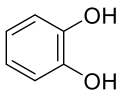

Catechol (19)

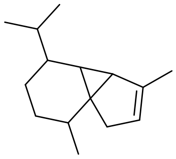

Alpha-cubebene (20)

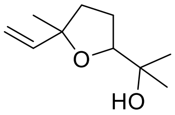

(E)-Linalool oxide (21)

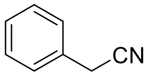

Benzeneacetonitrile (22)

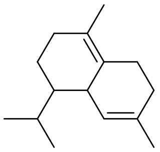

sigma-cadinene (23)

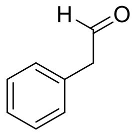

Phenylacetaldehyde (24)

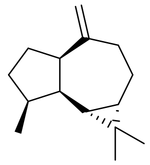

gamma-cadinene (25)

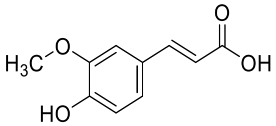

Ferulic acid (26)

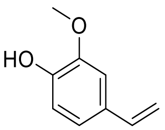

p-Vinylguaiacol (27)

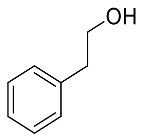

2-Phenylethyl alcohol (28)

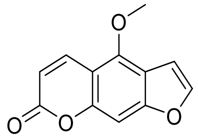

Bergapten (29)

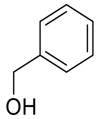

Benzyl alcohol **(30)**

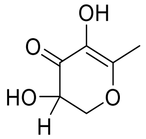

4H-Pyran-4-one,2,3-dihydro-3,5-dihydroxy-6-methyl (31)

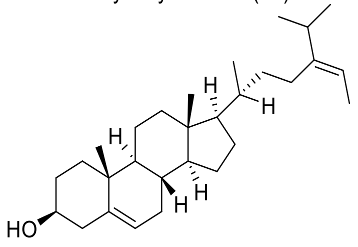

28-Isofucosterol (32)

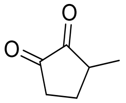

3-Methylcyclopenetane-1,2-dione (33)

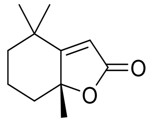

Dihydroactinidiolide (34)

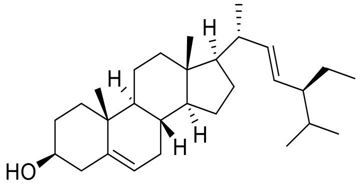

stigmasterol (35)

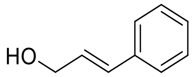

(E)-Cinnamyl alcohol (36)

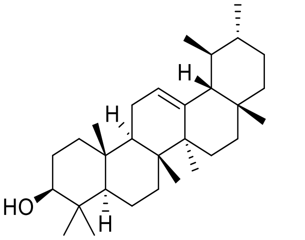

alpha-amyrin (37)

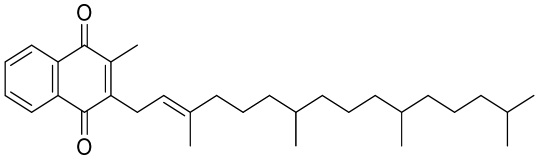

vitamin k1 (38)

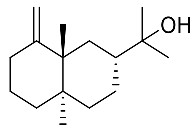

beta-eudesmol (39)

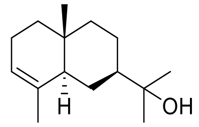

alpha-eudesmol (40)

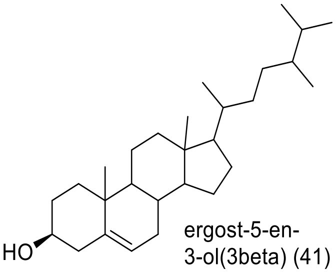

ergost-5-en-3-ol(3beta) (41)

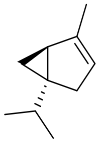

alpha- thujene (42)

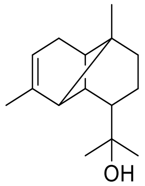

alpha-copaene-11-ol (43)

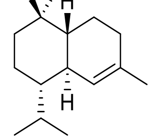

alpha-cadinol (44)

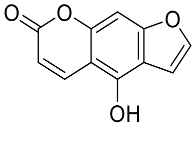

Bergaptol (45)

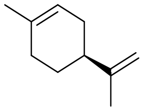

limonene (46)

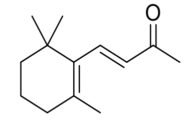

(E)-beta-Ionone (47)

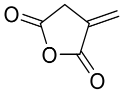

Itaconic anhydride (48)

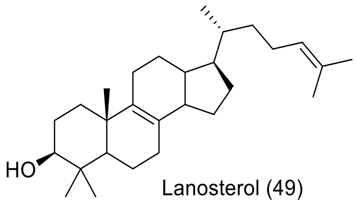

Lanosterol (49)

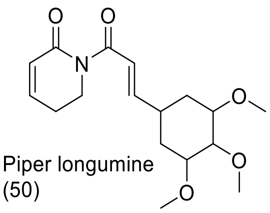

Piper longumine (50)

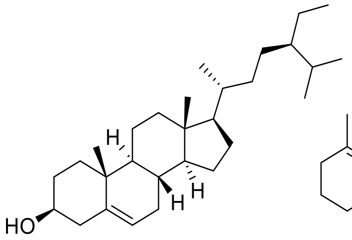

beta-sitosterol (51)

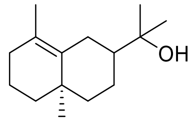

gamma-eudesmol (52)

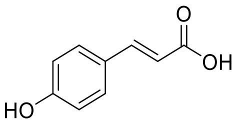

p-coumaric acid (53)

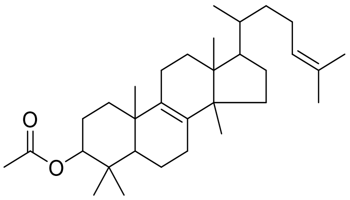

lanosta-8,24-dien-3-ol, acetate(3 beta) (54)

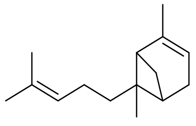

alpha-bergamotene (55)

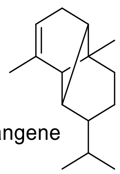

alpha-ylangene (56)

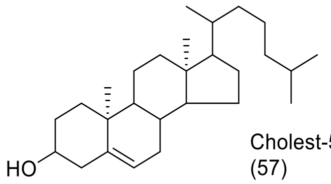

Cholest-5-en-3-ol (3 beta) (57)

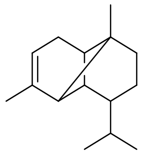

alpha-copaene (58)

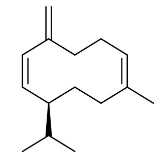

Germacrene-D (59)

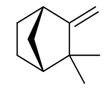

Camphene (60)

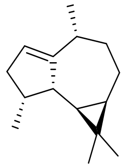

aromadendrene (61)

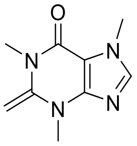

Caffeine (62)

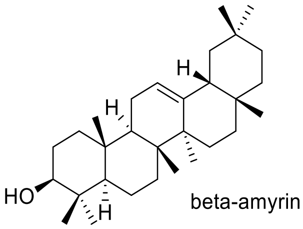

beta-amyrin (63)

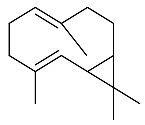

bicyclogermacrene (64)

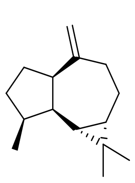

alloaromadendrene (65)

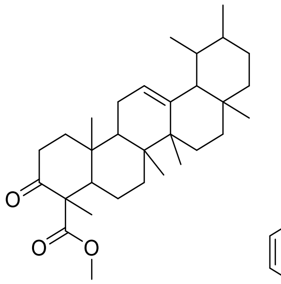

Urs-12-en-24-oic acid, 3-oxo-,methyl ester (66)

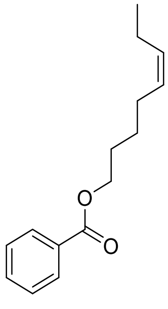

(3Z)-Hexenyl benzoate (67)

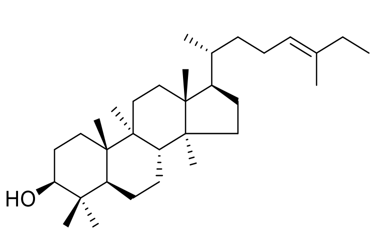

9,19-Cyclolanost_24-en-3-ol (3 beta) (68)

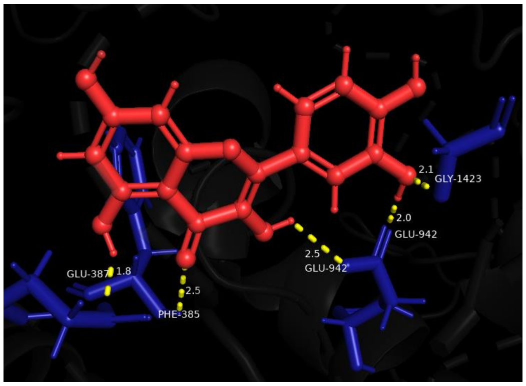

Figure S2 Quercetin in complex with $Na_v1.2$.

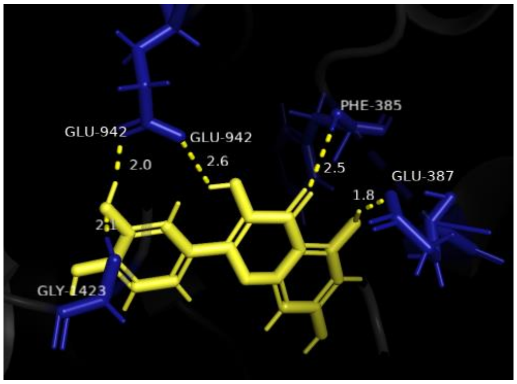

Figure S1 Methyl piperate in complex with $Na_v1.2$.

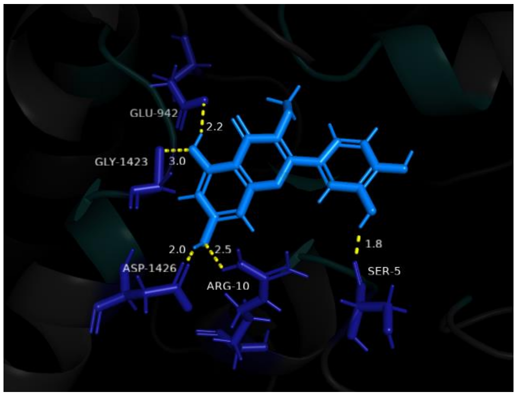

Figure S4 3,4,5,7-tetrahydroxy-3-methoxyflavone in complex with $Na_v1.2$.

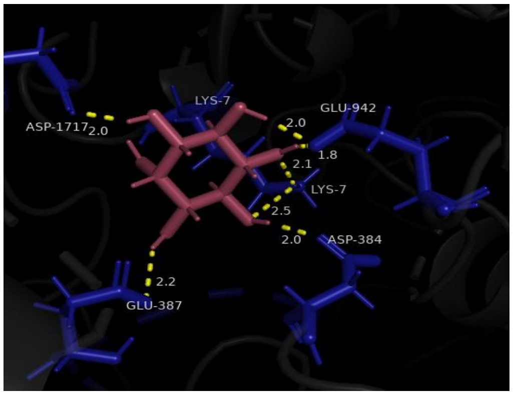

Figure S3 Inositol in complex with $Na_v1.2$.

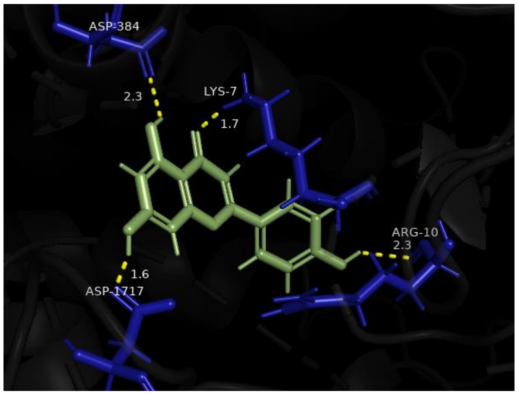

Figure S5 Apigenin in complex with $Na_v1.2$.

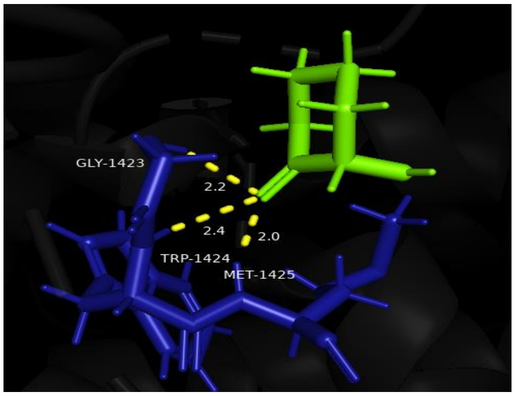

Figure S4 Adipoin in complex with $Na_v1.2$.

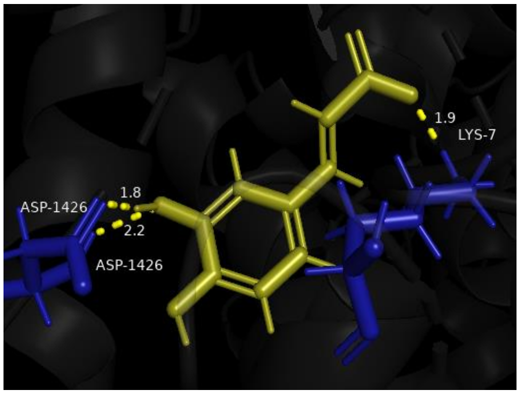

Figure S7 Caffeic acid in complex with Na_v1.2

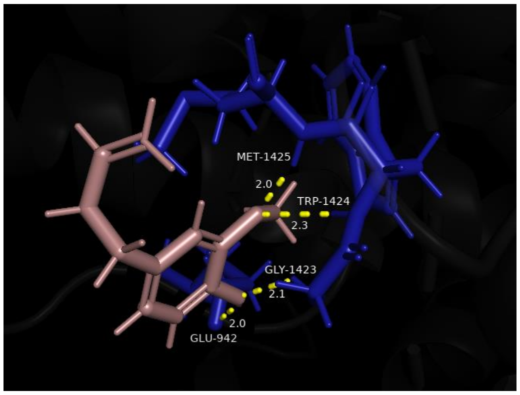

Figure S6 Eugenol in complex with Na_v1.2.

.

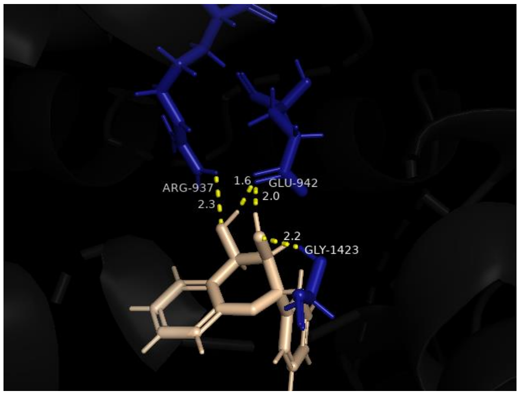

Figure S9 leucoanthocyanidin in complex with Na_v1.2.

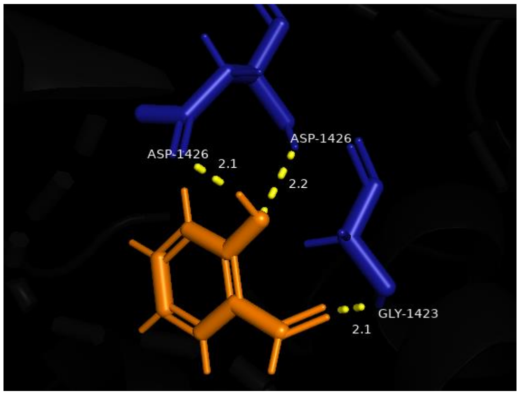

Figure S8 Salicylaldehyde in complex with Na_v1.2.

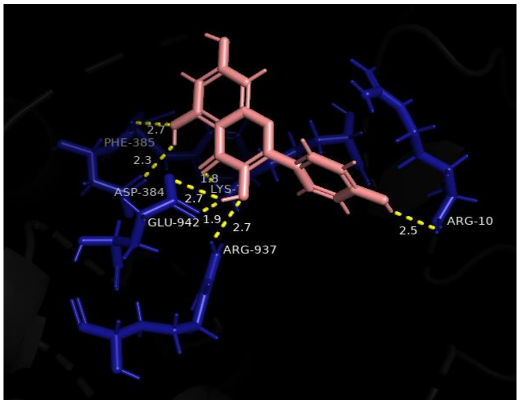

Figure S11 Kaempeferol in complex with Nav1.2

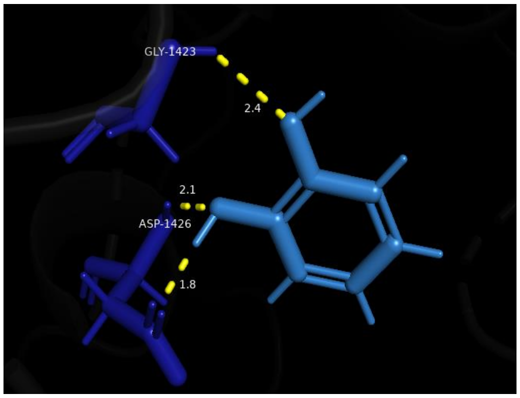

Figure S10 Catechol in complex with Na_v1.2.

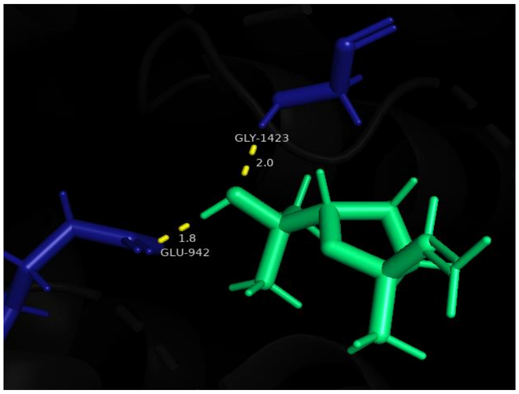

Figure S13 (E)-Linalool oxide in complex with $Na_v1.2$.

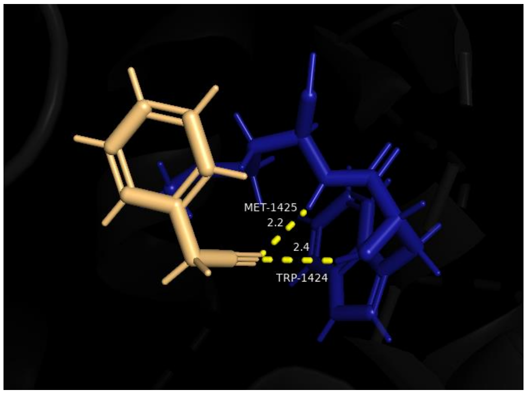

Figure S12 Benzeneacetonitrile in complex with $Na_v1.2$.

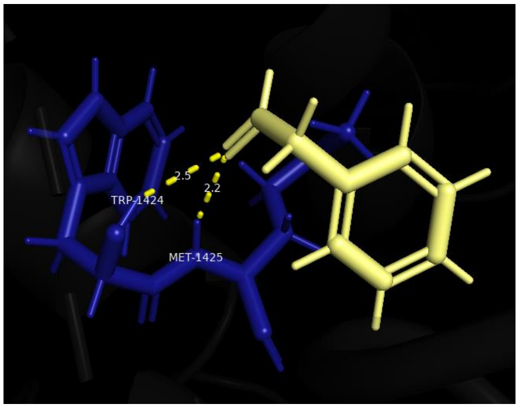

Figure S15 Phenylacetaldehyde in complex with $Na_v1.2$.

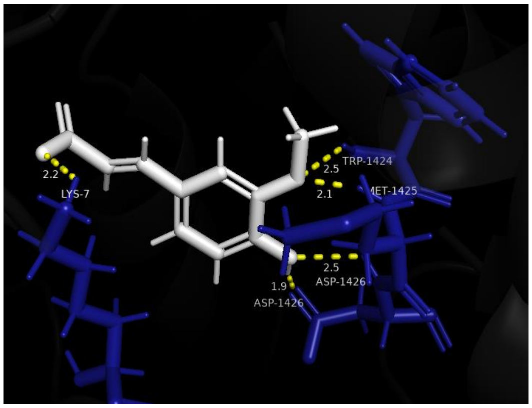

Figure S14 Ferulic acid in complex with $Na_v1.2$.

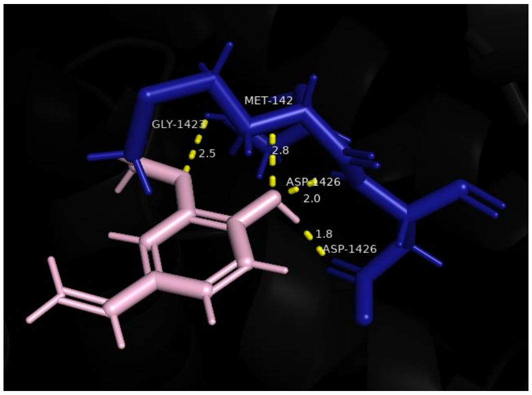

Figure S17 p-Vinylguaiacol in complex with $Na_v1.2$

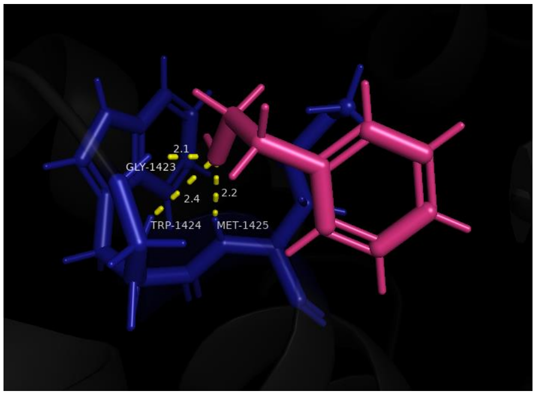

Figure S16 2-Phenylethyl alcohol in complex with $Na_v1.2$.

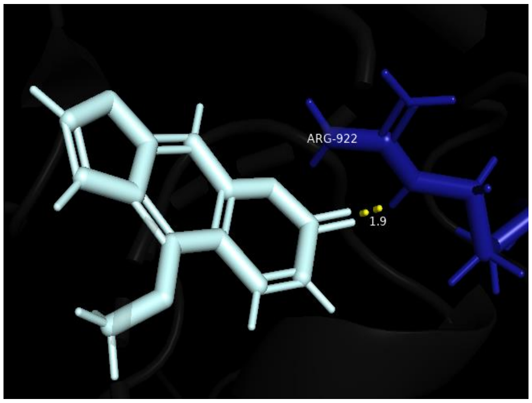

Figure S19 Bergapten in complex with Na_v1.2

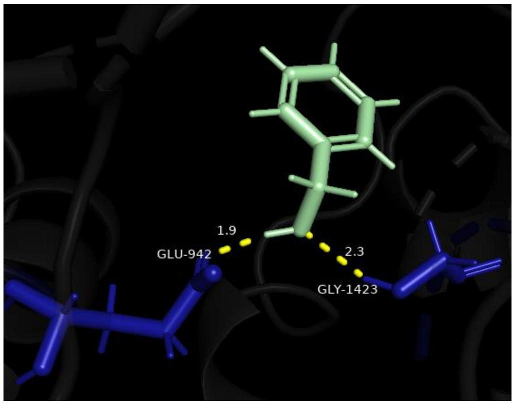

Figure S18 Benzyl alcohol in complex with Na_v1.2

.

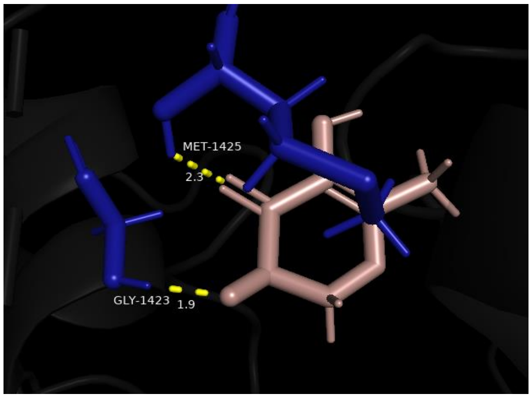

Figure S21 4H-Pyran-4-one,2,3-dihydro-3,5-dihydroxy-6-methyl in complex with Nav1.2.

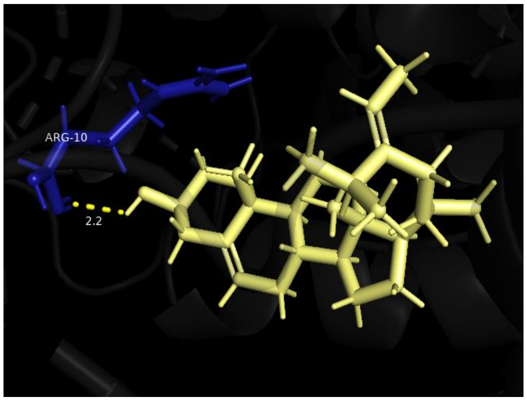

Figure S20 28-Isofucosterol in complex with Na_v1.2

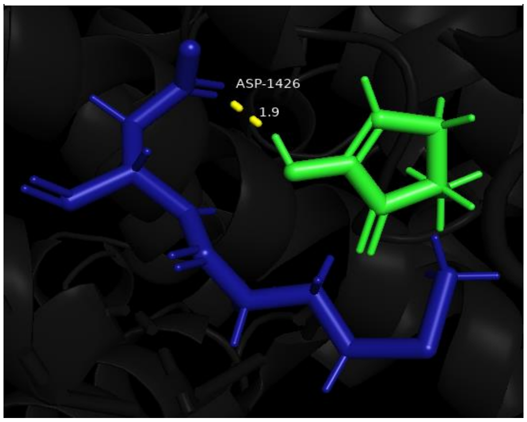

Figure S23 3-Methylcyclopenetane-1,2-dione in complex with Na_v1.2.

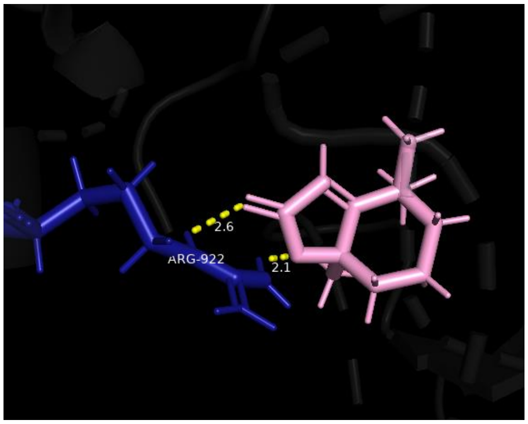

Figure S22 Dihydroactinidiolide in complex with Na_v1.2.

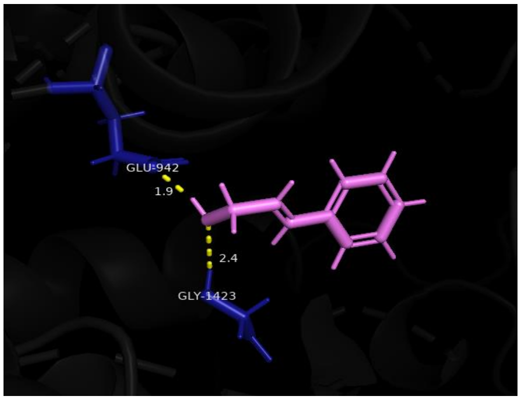

Figure S24 (E)-Cinnamyl alcohol in complex with $Na_v1.2$.

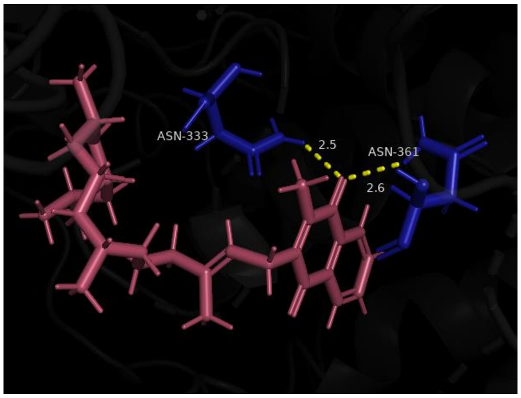

Figure S25 Vitamin k1 in complex with Nav1.2.

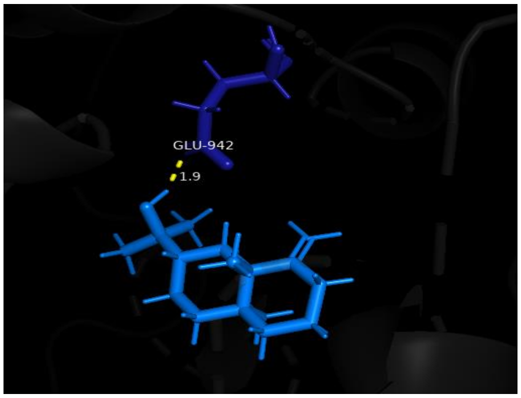

Figure S26 β-Eudesmol in complex with $Na_v1.2$.

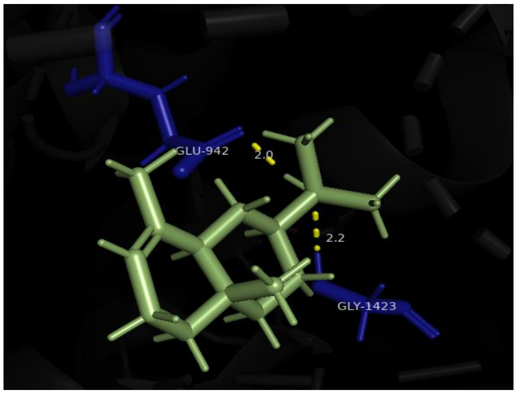

Figure S27 α-Eudesmol in complex with $Na_v1.2$.

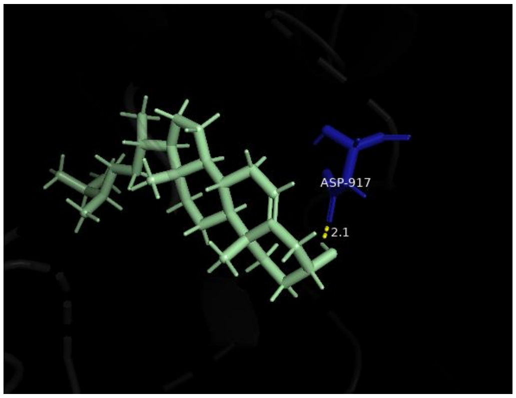

Figure S29 ergost-5-en-3-ol(3beta) in complex with $Na_v1.2$.

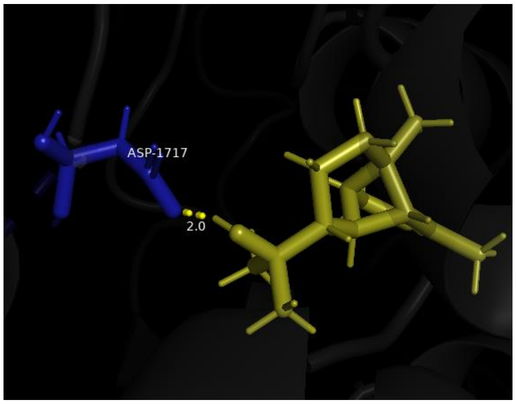

Figure S28 α-Copaene-11-ol in complex with $Na_v1.2$.

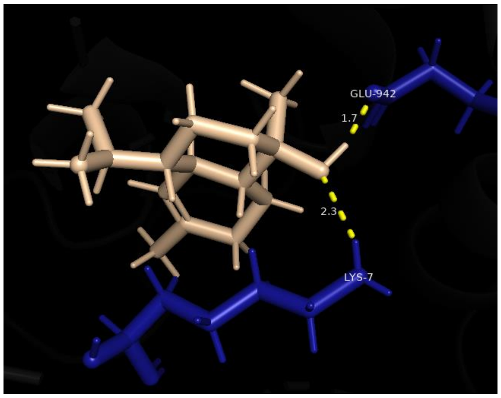

Figure S31 α-Cadinol in complex with Na_v1.2.

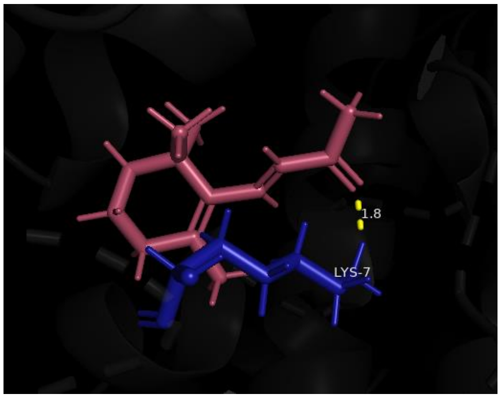

Figure S30 (E)-β-Ionone in complex with Na_v1.2.

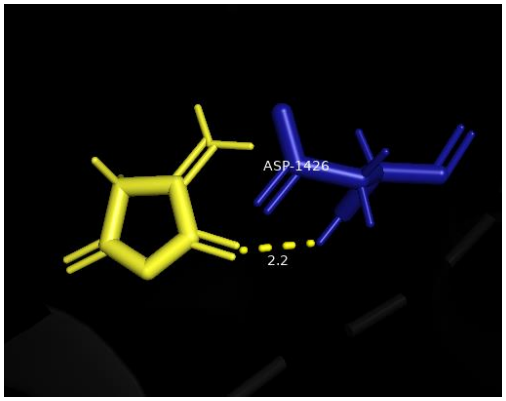

Figure S33 Itaconic anhydride in complex with Na_v1.2.

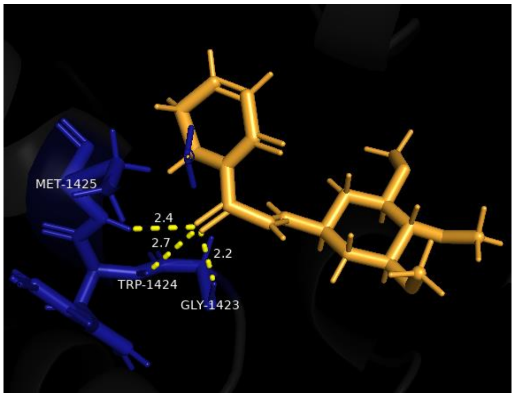

Figure S32 Piper longumine in complex with Na_v1.2.

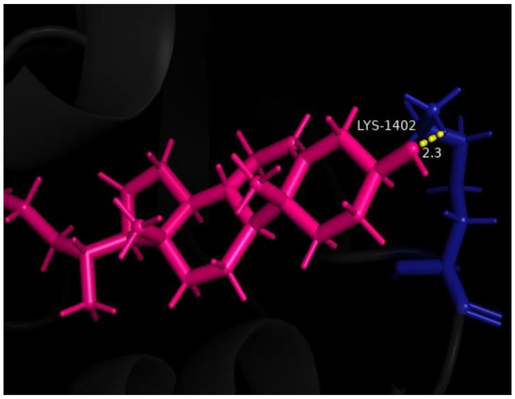

Figure S35 β-sitosterol in complex with Na_v1.2

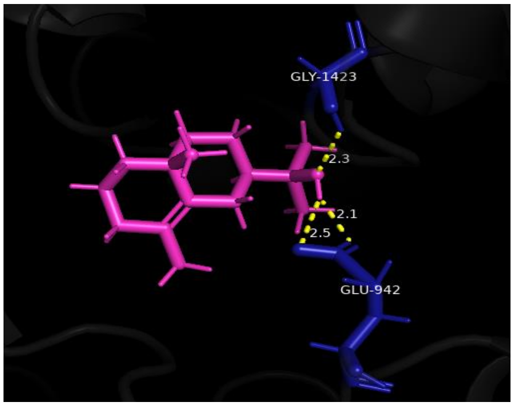

Figure S34 γ-Eudesmol in complex with Na_v1.2.

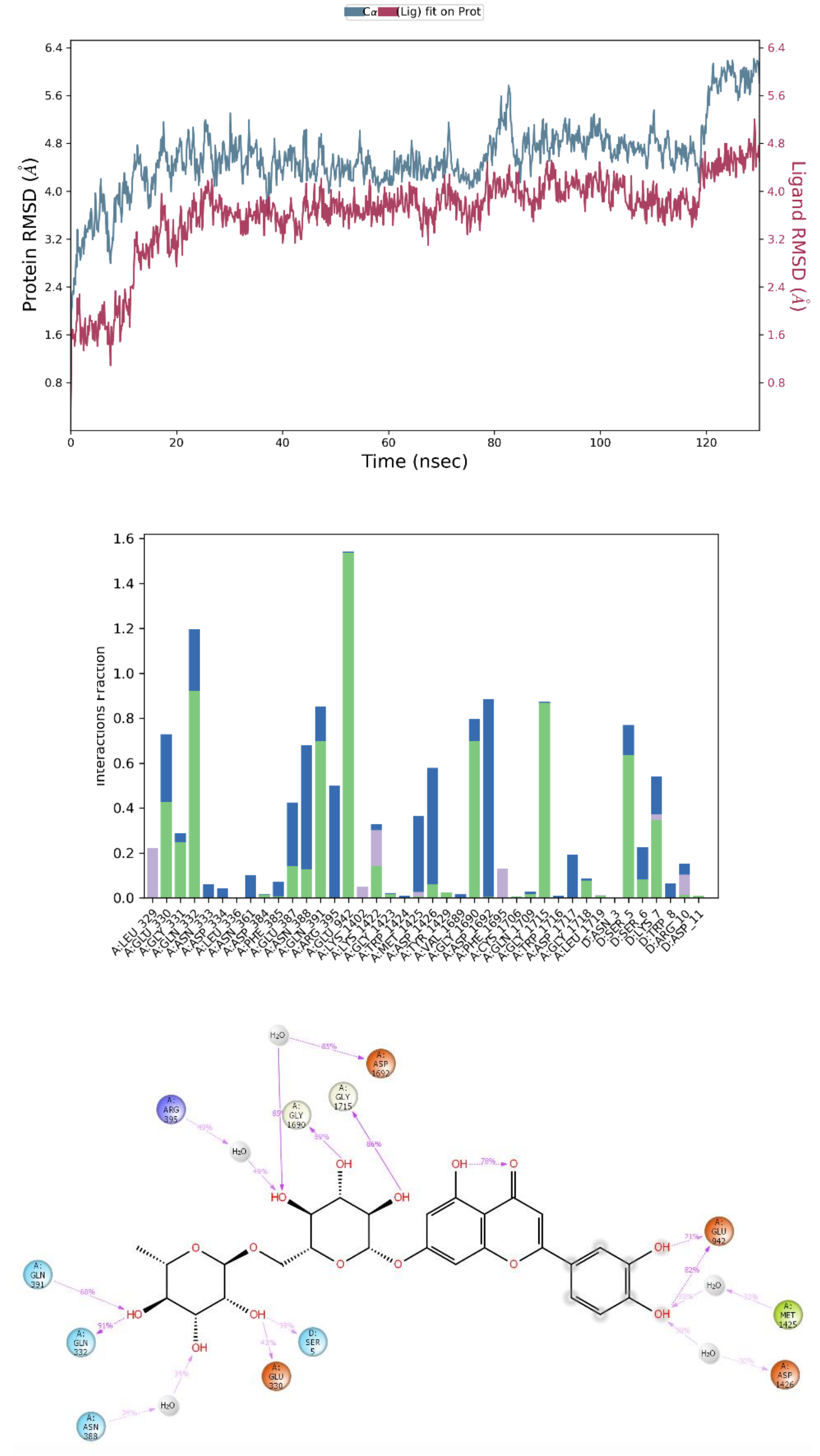

Figure S 38 MD simulation studies of luteolin 7-O-rutinoside in complex with $Na_v1.2$.

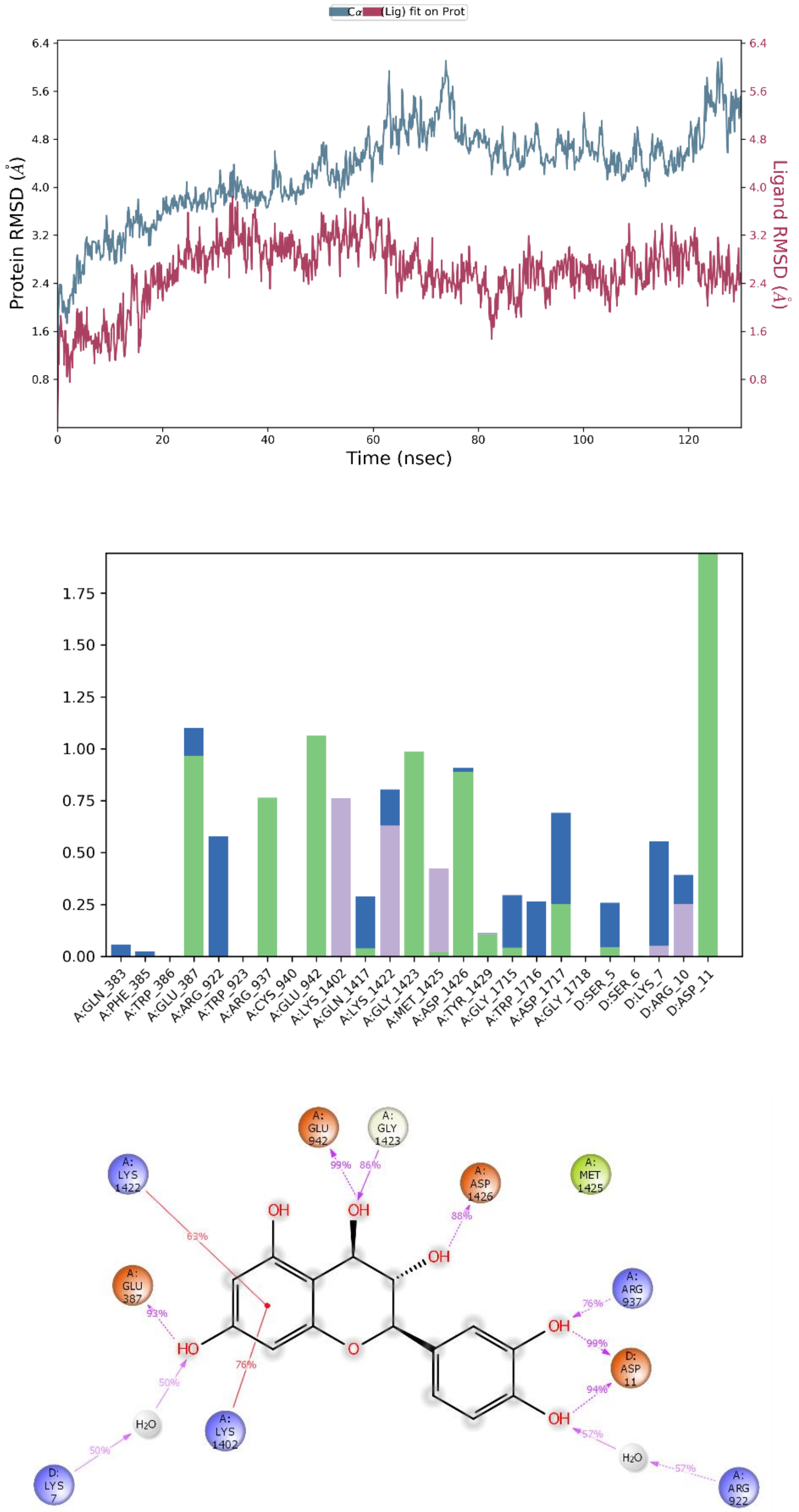

Figure S39 MD simulation studies of leucocyanidin in complex with $Na_v1.2$.
